# Supplementary material for: Structural basis of THC analog activity at the Cannabinoid 1 receptor
Source: Nat Commun. 2025 Jan 8;16:486. doi: 10.1038/s41467-024-55808-4 (PMC11711184; doi:10.1038/s41467-024-55808-4)
Supplement: Supplementary file 1 — Supplementary Information [file 41467_2024_55808_MOESM1_ESM.pdf]

# SUPPLEMENTARY INFORMATION

for

**Structural basis of THC analog activity at the Cannabinoid 1 receptor**

Thor S. Thorsen[1,8,10], Yashraj Kulkarni[1,10], David A. Sykes[2,3], Andreas Bøggild[4], Taner Drace[4], Pattarin Hompluem[2,3], Christos Iliopoulos-Tsoutsouvas[5], Spyros P. Nikas[5], Henrik Daver[1,9], Alexandros Makriyannis[5,6], Poul Nissen[4,7], Michael Gajhede[1], Dmitry B. Veprintsev[2,3,11], Thomas Boesen[4,11], Jette S. Kastrup[1,11] and David E. Gloriam[1,11*]

*[1] Department of Drug Design and Pharmacology, University of Copenhagen, Universitetsparken 2, 2100 Copenhagen, Denmark*
*[2] Centre of Membrane Proteins and Receptors (COMPARE), University of Nottingham, Midlands NG7 2RD, UK.*
*[3] Division of Physiology, Pharmacology & Neuroscience, School of Life Sciences, University of Nottingham, Nottingham NG7 2UH, UK.*
*[4] Department of Molecular Biology & Genetics, Aarhus University, Aarhus, Denmark*
*[5] Center for Drug Discovery and Department of Pharmaceutical Sciences, Northeastern University, Boston, Massachusetts 02115, United States.*
*[6] Center for Drug Discovery and Departments of Chemistry and Chemical Biology, Northeastern University, Boston, Massachusetts 02115, United States.*
*[7] Aarhus University, Dept. Molecular Biology and Genetics, Danish Research Institute of Translational Neuroscience – DANDRITE, NordicEMBL Partnership for Molecular Medicine, Denmark*
*[8] Present address: Nordic Virtual Pastures, BioInnovation Institute, Ole Maaløes Vej 3, 2200 København N, Denmark*
*[9] Present address: H. Lundbeck A/S, Ottiliavej 9, 2500 Valby, Denmark*
*[10] These authors contributed equally to the work.*
*[11] These authors have jointly supervised the work.*
**Correspondence: david.gloriam@sund.ku.dk (D.E.G.)*

## Supplementary Figures

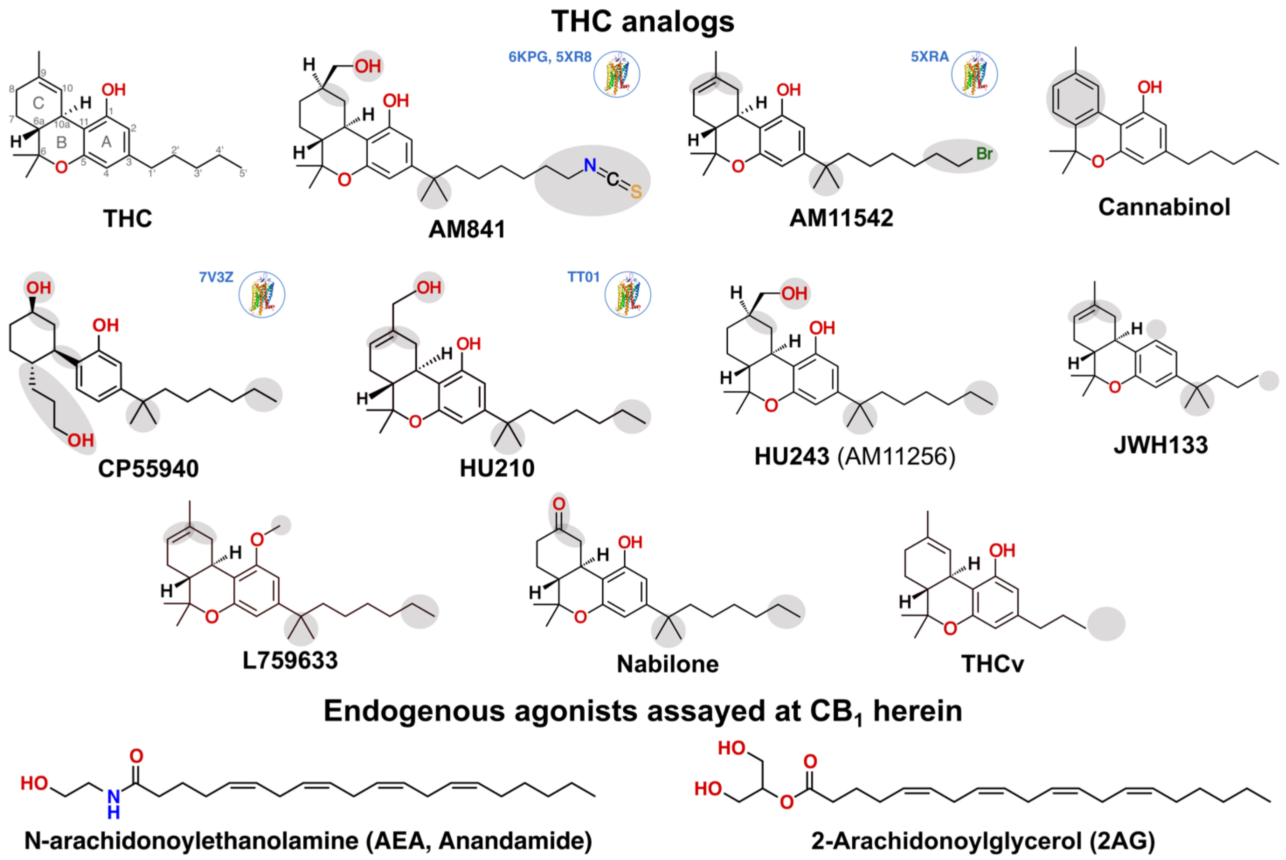


**Supplementary Fig. 1 | Chemical structures of THC analogs and additional agonists assayed at $CB_1$.** A structure icon and PDB identifiers denote ligands with experimental structures.

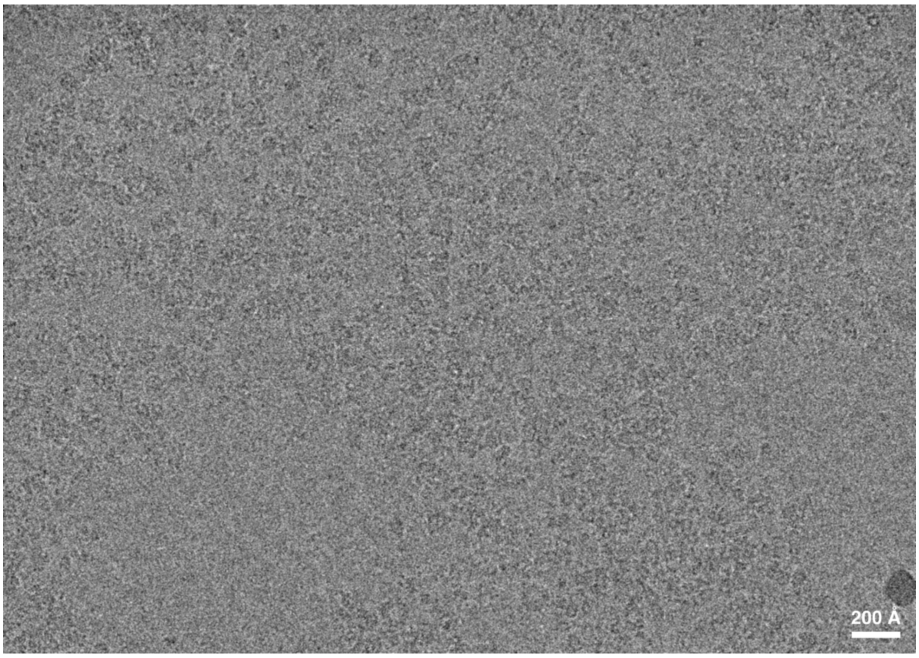


**Supplementary Fig. 2 | Raw cryo-EM micrograph.**

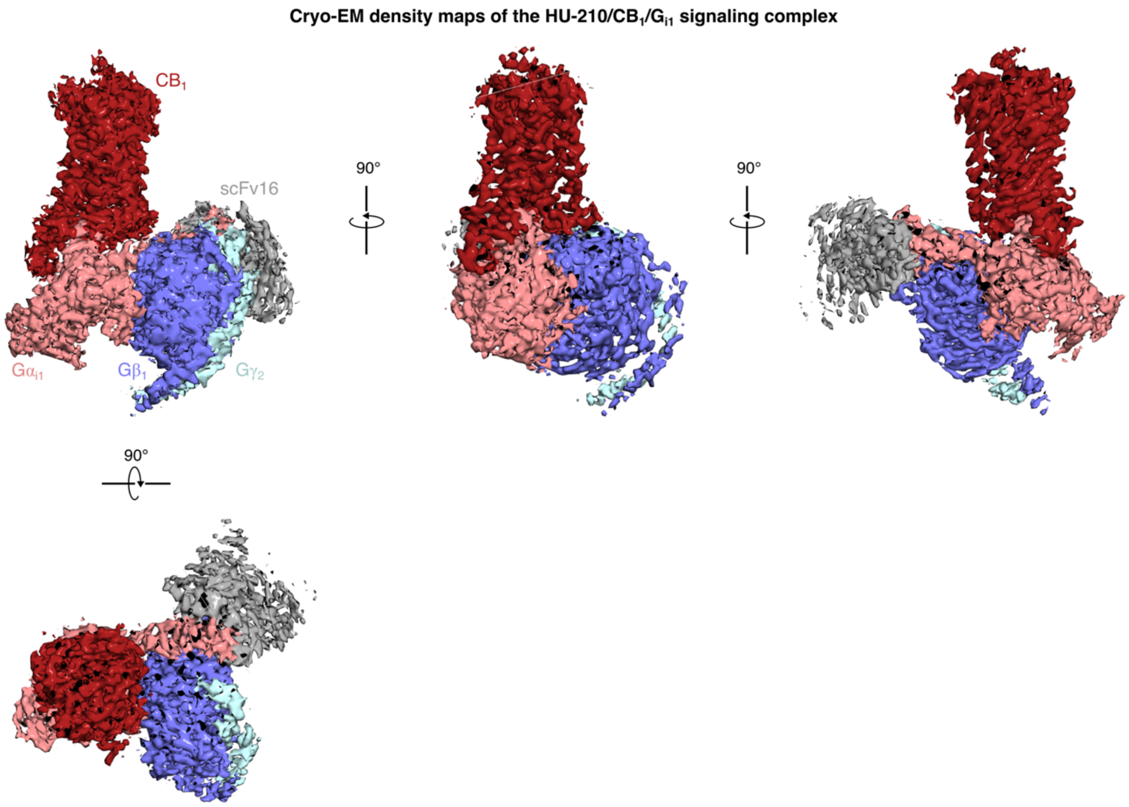


**Supplementary Fig. 3 | Cryo-EM density map (sharpened) of the HU-210/CB$_1$/G$_{i1}$ signaling complex.** Additional viewing angles (horizontally and vertically rotated) of the HU-210/CB$_1$/G$_{i1}$ signaling complex taking Fig. 1a as the reference view.

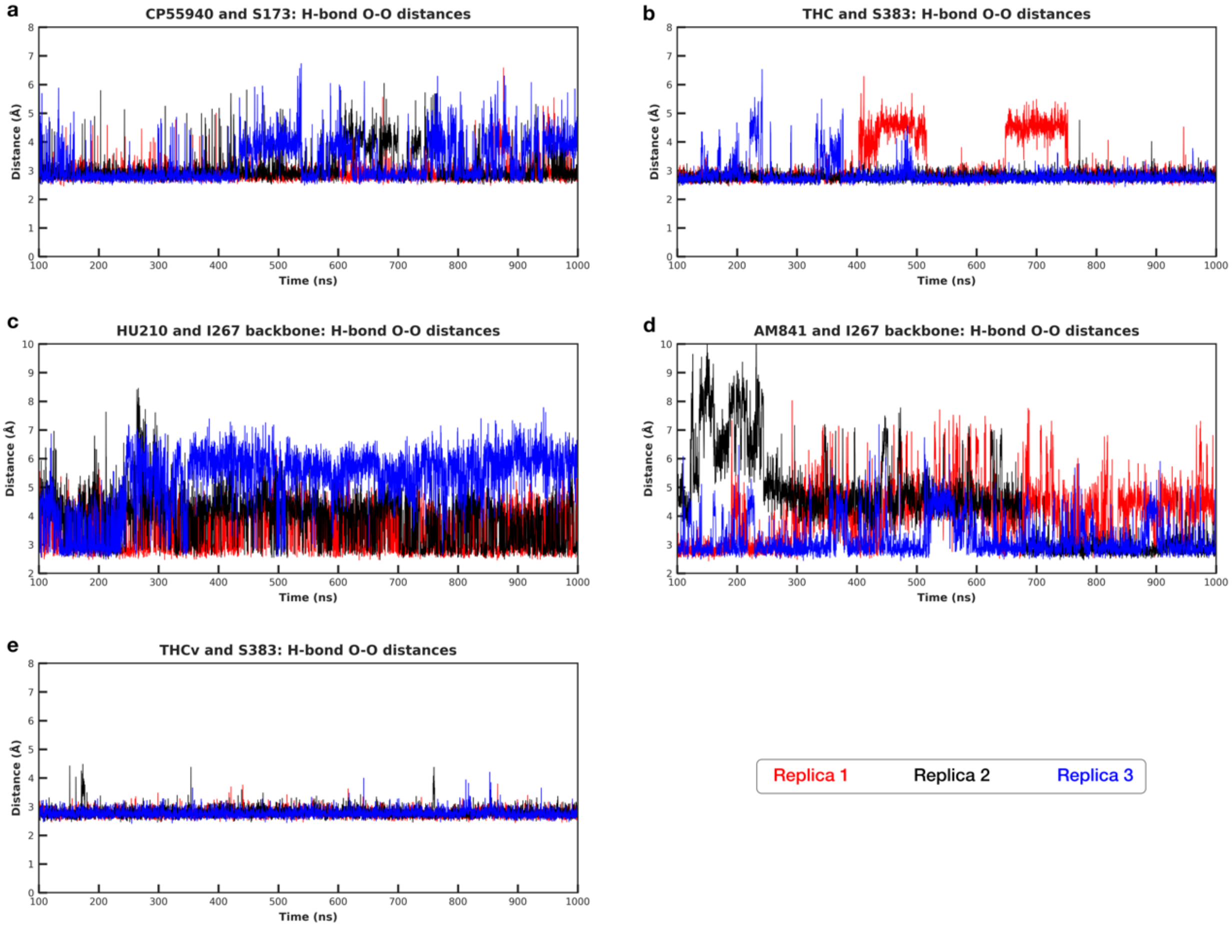


**Supplementary Fig. 4 | Distances between hydrogen bonding heavy atoms.** Time-resolved distances between heavy atoms participating in a hydrogen bond as indicated in Fig. 5 in the main text. **a,** 6a hydroxypropyl of CP55940 and sidechain hydroxyl of $S173^{2x60}$. **b,** C1 hydroxyl of THC and sidechain hydroxyl of $S383^{7x38}$. **c-d,** C9 hydroxymethyl of HU210 and AM841 respectively, and backbone oxygen of $I267^{ECL2}$. **e,** C1 hydroxyl of THCv and sidechain hydroxyl of $S383^{7x38}$. The first 100 ns have been omitted in the plots as part of system equilibration. Data within each plot correspond to individual replicates (Red: Replica 1, Black: Replica 2, Blue: Replica 3).

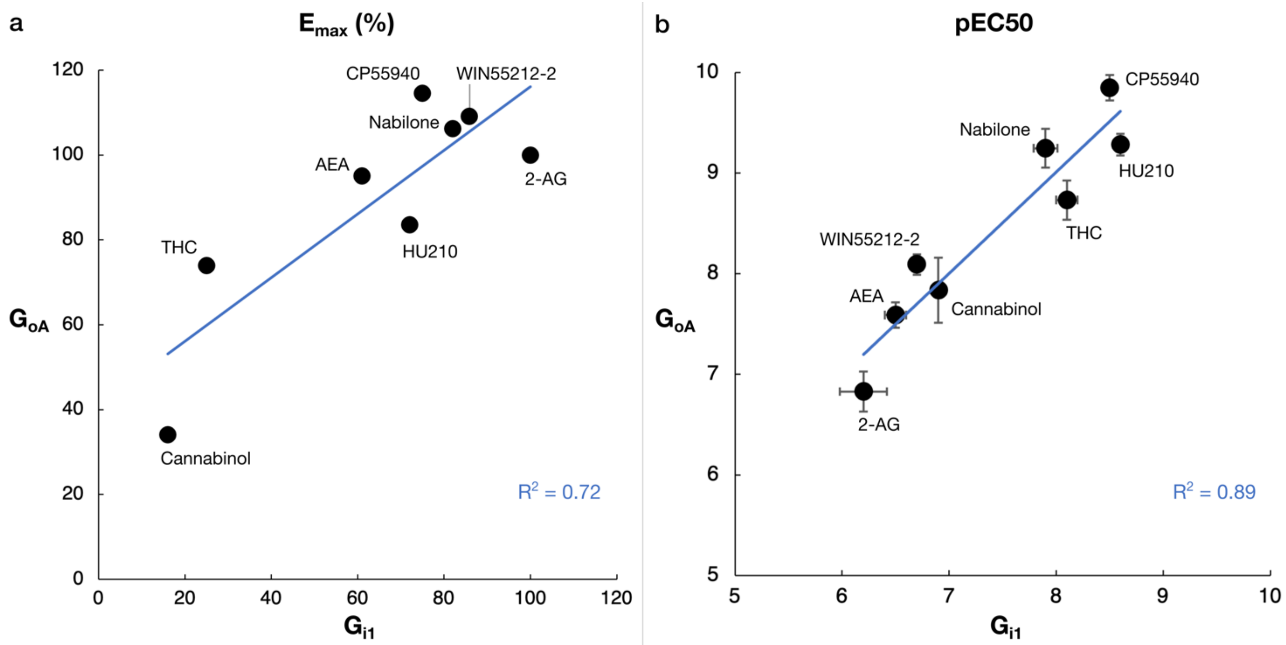


**Supplementary Fig. 5 | Correlation of $G_{i1}$ and $G_{oA}$ recruitment for different $CB_1$ agonists. a,** Scatter plot of $E_{max}$ values, $G_{i1}$ and $G_{oA}$ recruitment. **b,** Scatter plot of $pEC_{50}$ values, $G_{i1}$ versus $G_{oA}$ recruitment. The $R^2$ values show the coefficient of determination for linear correlations. Data corresponding to $G_{oA}$ are from our previous study[1] and were produced using a BRET-based G protein-recruitment assay which employs Renilla luciferase RlucII-fused signaling effectors and energy acceptors that are anchored to the plasma membrane (rGFP-CAAX).

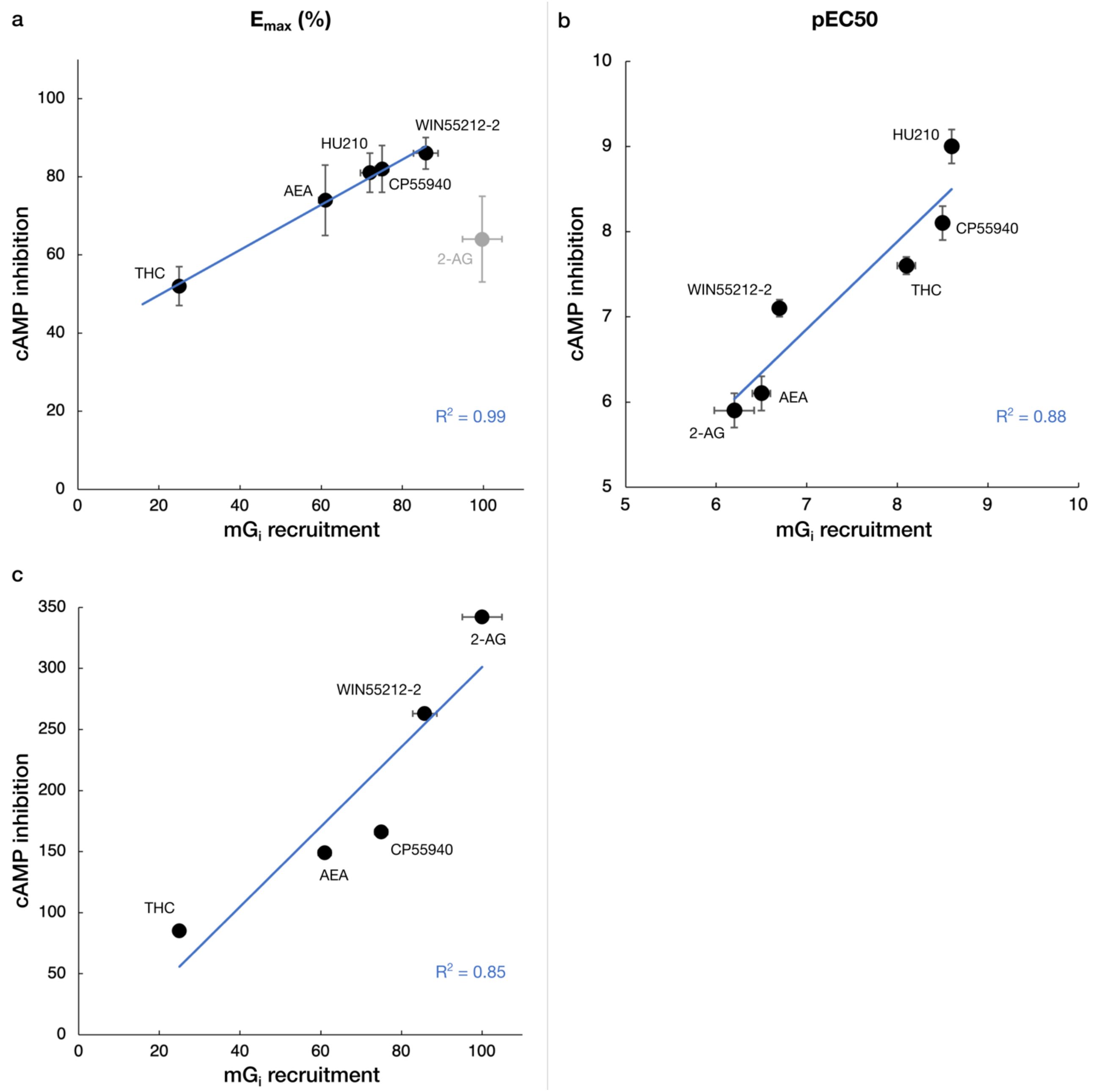


**Supplementary Fig. 6 | Correlation of mG$_i$ recruitment and cAMP inhibition for different CB$_1$ agonists.** **a,c** Scatter plots of E$_{max}$ values, G$_{i1}$ recruitment versus cAMP inhibition. **b,** Scatter plot of pEC$_{50}$ values, G$_{i1}$ recruitment versus cAMP inhibition. Efficacy data is calculated using 2AG as a reference in our current study for G$_{i1}$ recruitment, and forskolin stimulation in [2] for cAMP inhibition. The $R^2$ values show the coefficient of determination for linear correlations. G$_{i1}$ recruitment data are from the current study whereas cAMP inhibition data are taken from the literature [2] for **a,b** and [3] for **c**. The cAMP inhibition efficacy data taken from the literature[2], as shown in **a**, show excellent correlation with data from our current study. However, 2AG stands out as an outlier from this linear correlation, as shown in **c** that also contains cAMP inhibition data taken from another literature source [3] demonstrating that 2AG has the highest efficacy among the ligands tested, a finding consistent with its performance in our BRET-based Gi1 recruitment experiments.

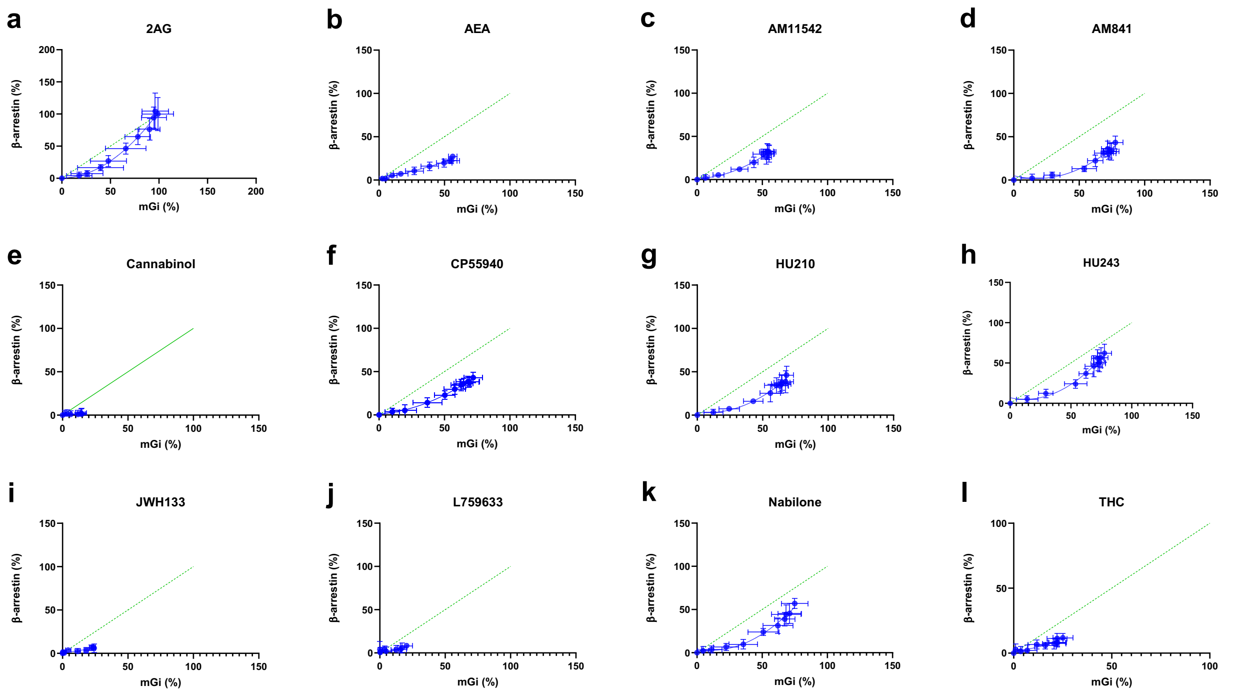


**Supplementary Fig. 7 | Bias plots for THC analogs and endogenous agonists.** $mG_i$ and β-arrestin recruitment at equimolar ligand concentrations expressed as percent of the maximal response elicited by the endogenous cannabinoid 2AG. The green dotted line depicts a (theoretical) pathway-balanced ligand. Data is shown as mean ± S.E.M. of at least three independent experiments and were fitted to a second-order polynomial.

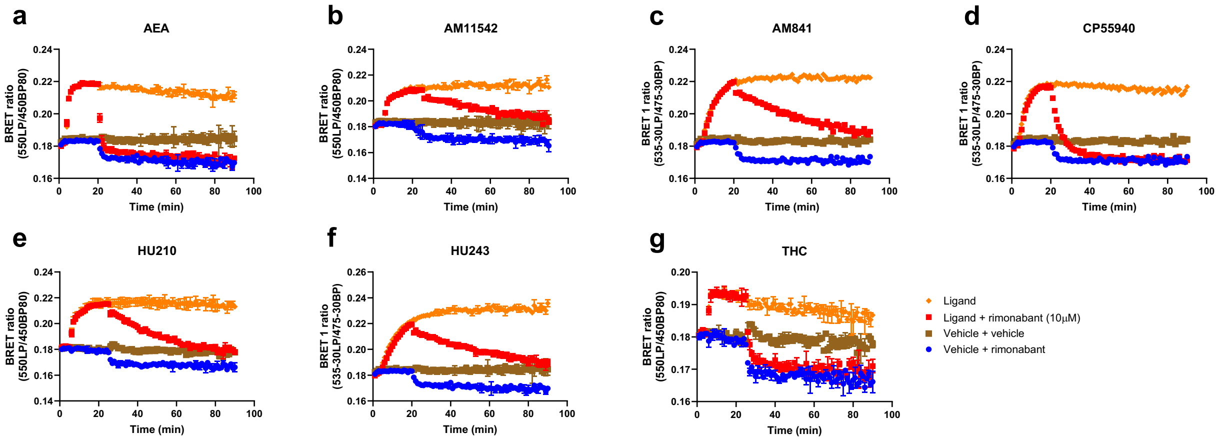


**Supplementary Fig. 8 | Recruitment of mG$_i$ by agonists and its reversal upon addition of the CB$_1$ specific antagonist rimonabant.** HEK293-TR cells stably expressing CB1R-NlucC and NES-venus-mGsi, stimulated with an EC$_{80}$ concentration of the agonists AEA (**a**), AM11542 (**b**), AM841 (**c**), CP55940 (**d**), HU210 (**e**), HU243 (**f**), and THC (**g**). The change in basal BRET is plotted versus time, mG$_i$ recruitment reversal was initiated by the addition of an excess concentration of the CB$_1$-specific antagonist rimonabant (10 μM). mG$_i$ responses are shown as mean ± S.D. and are representative of at least three independent experiments performed in duplicate.

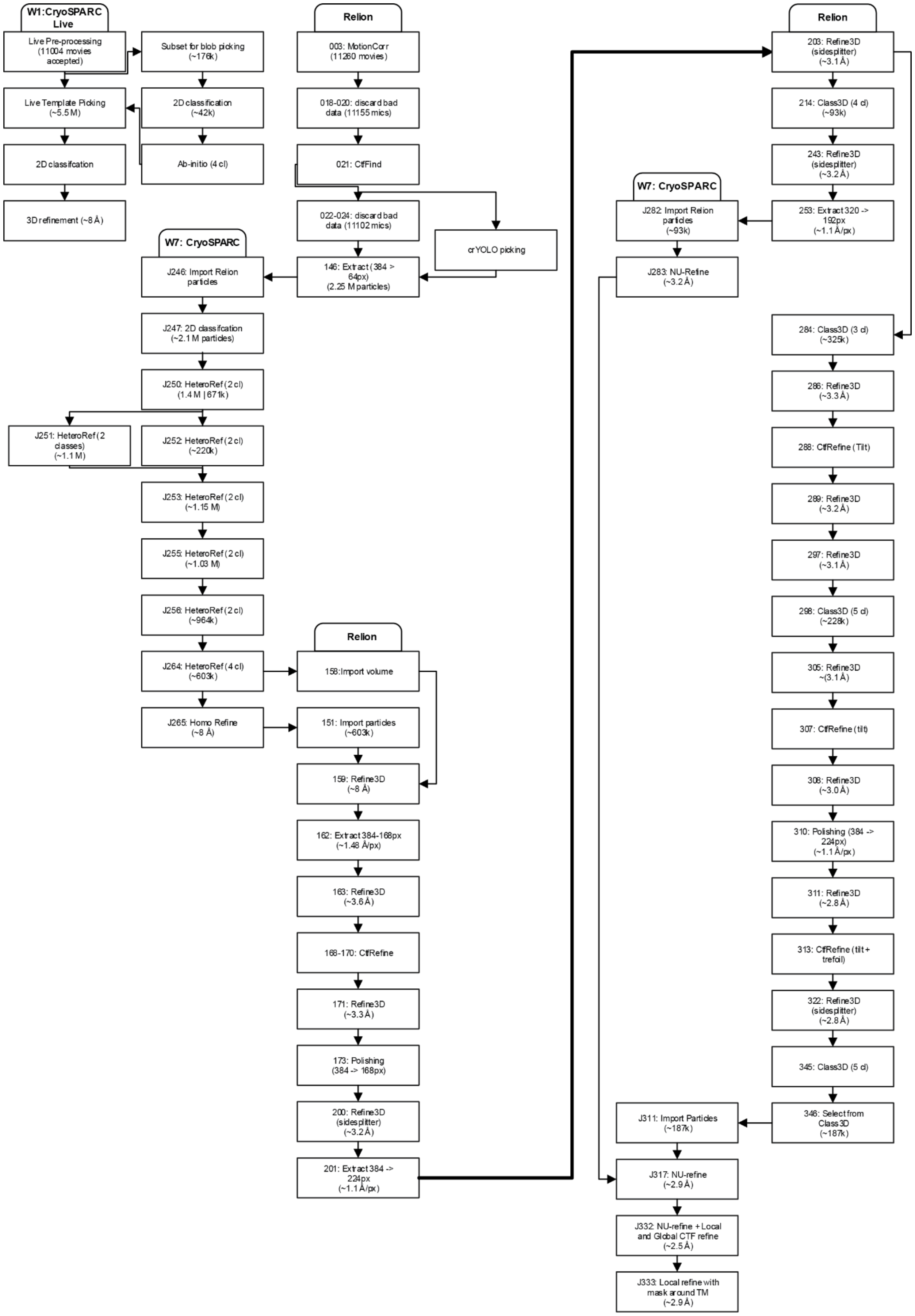
W1:CryoSPARC Live
Live Pre-processing (11004 movies accepted)
Subset for blob picking (~176k)
Live Template Picking (~5.5 M)
2D classification (~42k)
2D classifcation
Ab-initio (4 cl)
3D refinement (~8 Å)
Relion
003: MotionCorr (11260 movies)
018-020: discard bad data (11155 mics)
021: CtfFind
022-024: discard bad data (11102 mics)
crYOLO picking
146: Extract (384 > 64px) (2.25 M particles)
W7: CryoSPARC
J246: Import Relion particles
J247: 2D classifcation (~2.1 M particles)
J250: HeteroRef (2 cl) (1.4 M | 671k)
J251: HeteroRef (2 classes) (~1.1 M)
J252: HeteroRef (2 cl) (~220k)
J253: HeteroRef (2 cl) (~1.15 M)
J255: HeteroRef (2 cl) (~1.03 M)
J256: HeteroRef (2 cl) (~964k)
J264: HeteroRef (4 cl) (~603k)
J265: Homo Refine (~8 Å)
Relion
158: Import volume
151: Import particles (~603k)
159: Refine3D (~8 Å)
162: Extract 384-168px (~1.48 Å/px)
163: Refine3D (~3.6 Å)
168-170: CtfRefine
171: Refine3D (~3.3 Å)
173: Polishing (384 -> 168px)
200: Refine3D (sidesplitter) (~3.2 Å)
201: Extract 384 -> 224px (~1.1 Å/px)
Relion
203: Refine3D (sidesplitter) (~3.1 Å)
214: Class3D (4 cl) (~93k)
243: Refine3D (sidesplitter) (~3.2 Å)
253: Extract 320 -> 192px (~1.1 Å/px)
W7: CryoSPARC
J282: Import Relion particles (~93k)
J283: NU-Refine (~3.2 Å)
284: Class3D (3 cl) (~325k)
286: Refine3D (~3.3 Å)
288: CtfRefine (Tilt)
289: Refine3D (~3.2 Å)
297: Refine3D (~3.1 Å)
298: Class3D (5 cl) (~228k)
305: Refine3D ~(3.1 Å)
307: CtfRefine (tilt)
308: Refine3D (~3.0 Å)
310: Polishing (384 -> 224px) (~1.1 Å/px)
311: Refine3D (~2.8 Å)
313: CtfRefine (tilt + trefoil)
322: Refine3D (sidesplitter) (~2.8 Å)
345: Class3D (5 cl)
346: Select from Class3D (~187k)
J311: Import Particles (~187k)
J317: NU-refine (~2.9 Å)
J332: NU-refine + Local and Global CTF refine (~2.5 Å)
J333: Local refine with mask around TM (~2.9 Å)

**Supplementary Fig. 9 | Schematic overview of processing workflow, showing key jobs from Relion and Cryosparc.** Cryosparc Live was used to monitor the data collection and get an initial idea about data quality. Processing was then started from scratch in Relion with motion correction and CTF estimation, followed by crYOLO picking. The particles were imported to Cryosparc for 2D classification and Heterogeneous Refinement runs to remove bad particles. The new stack was imported back into Relion for multiple runs of 3D refinement (with and without using Sidesplitter), CTF refinements, 3D classifications and Polishing. Particle binning was reduced through the iterations as resolution improved. The refined stack was imported back into Cryosparc for Non-uniform refinement, Global and Local CTF refinements and finally a Local refinement with a mask around the trans-membrane domain.

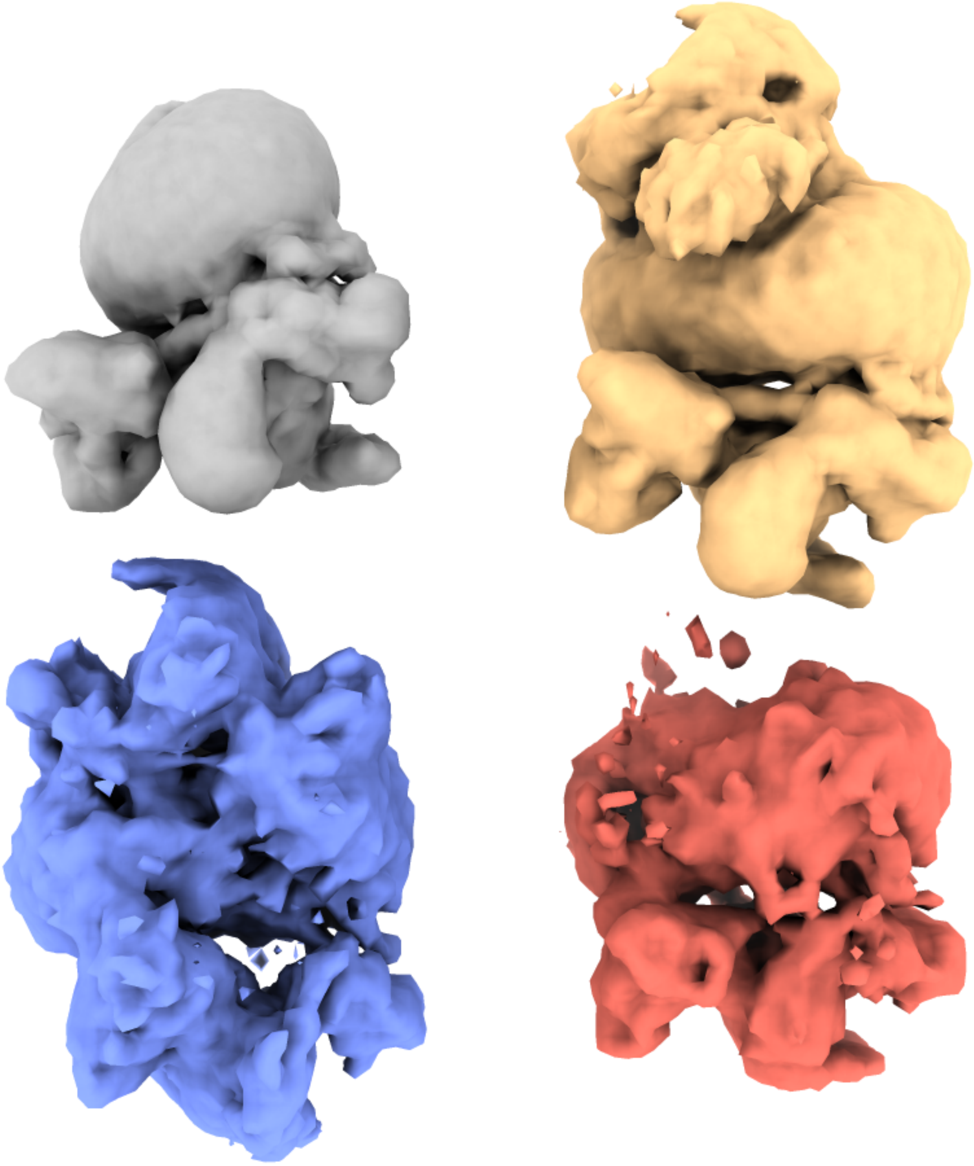

**Supplementary Fig. 10 | Heterogenous Refinement.** Four classes from a Heterogeneous Refinement job in Cryosparc (J264 in the schematic workflow overview, Supplementary Fig. 9). From ~964k particles in the input stack, the good class above (grey) ended up with ~603k. The junk classes ended up with ~174k (yellow), ~92k (blue) and ~95k (red) particles. Input volumes for junk classes were *ab initio* generated from a selection of 2D classes showing two complexes integrated into one micelle.

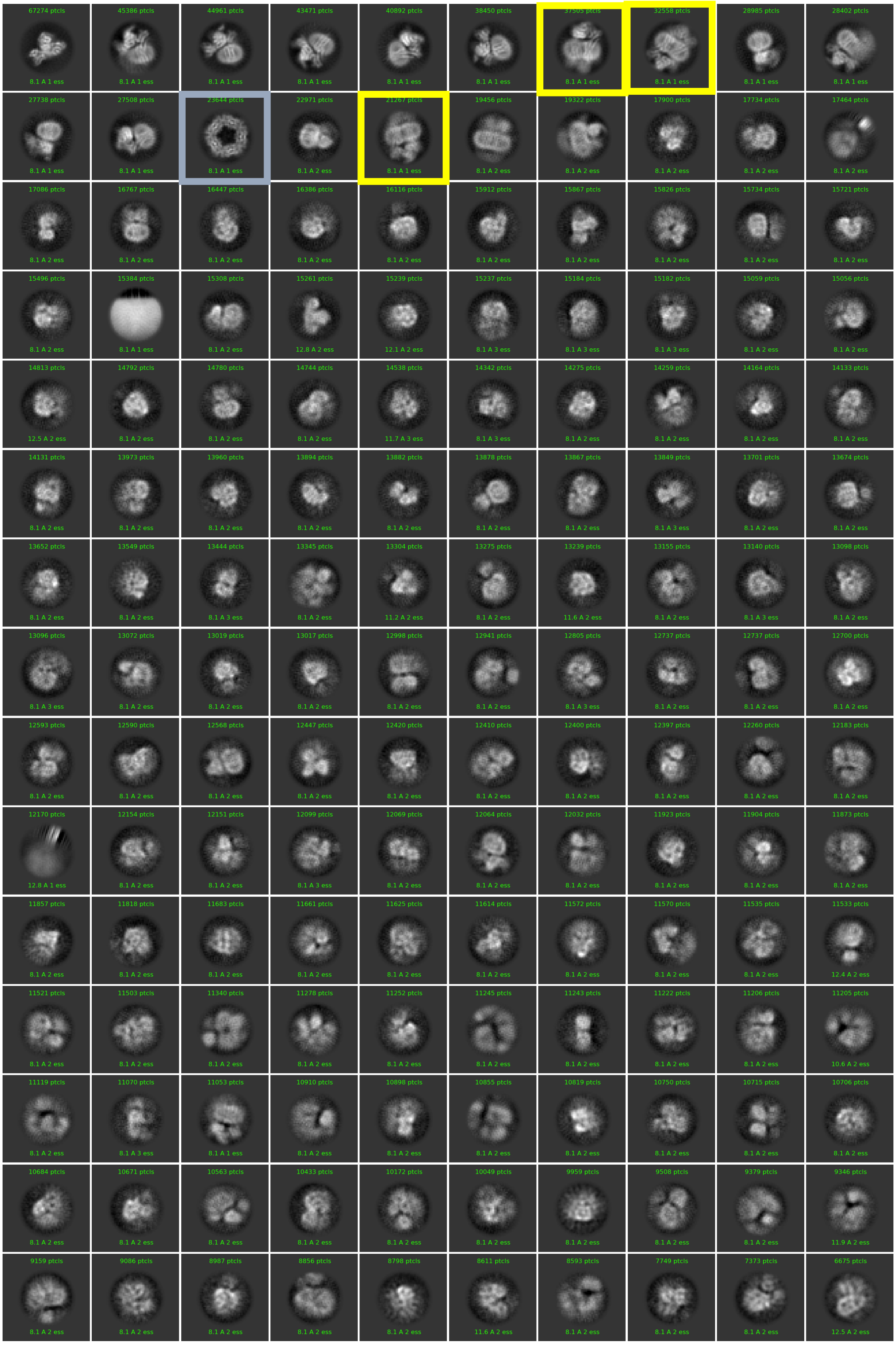

**Supplementary Fig. 11 | 2D classification.** Early classification showing many junk classes, including an unidentified protein contaminant (blue) and particles with two complexes in one shared micelle (yellow) – J247 in the schematic workflow overview (Supplementary Fig. 9).

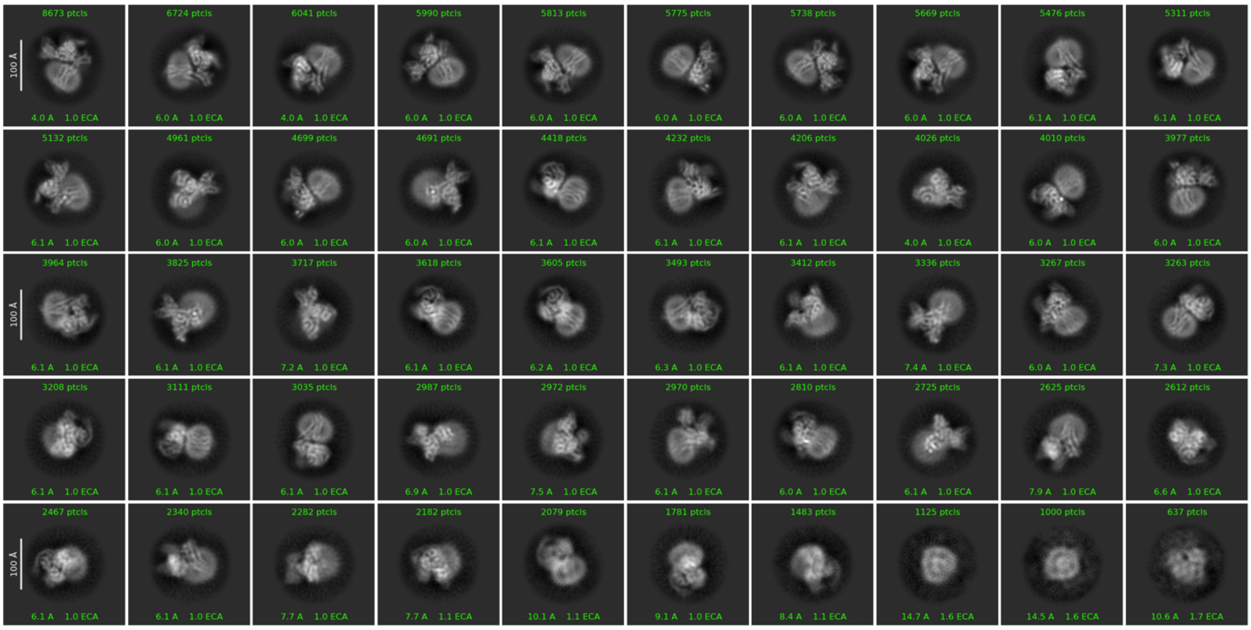


**Supplementary Fig. 12 | 2D classification of the particle stack used in the final 3D reconstruction.**

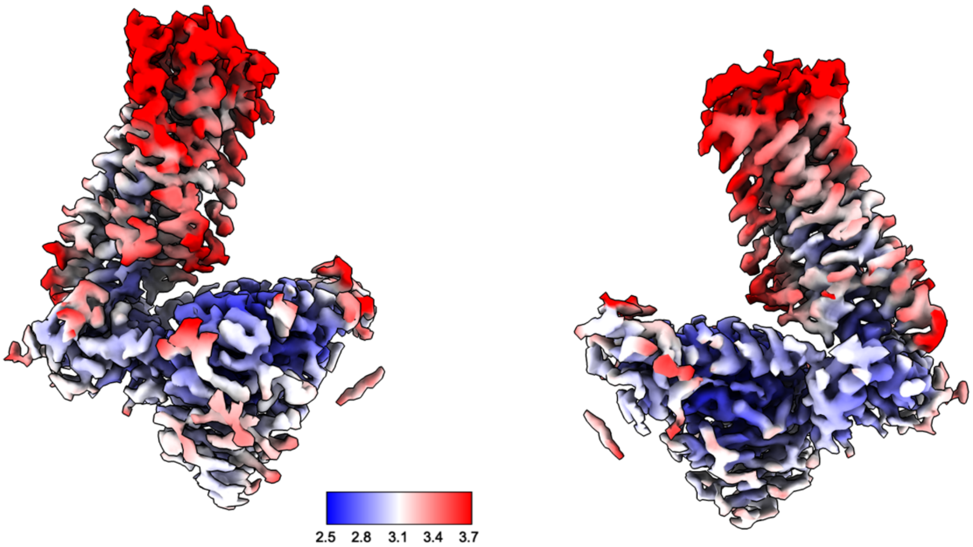


**Supplementary Fig. 13 | Local resolution estimation.** From Cryosparc v4.6.2 Local Resolution using a Blocres-like algorithm[4] on the final Local Refinement (focus mask on TM) half-maps (J333 in Supplementary Fig. 6). Visualized in ChimeraX and scaled to highlight the TM-region. Scale in Å.

Sampling Compensation Factor (SCF)[5]

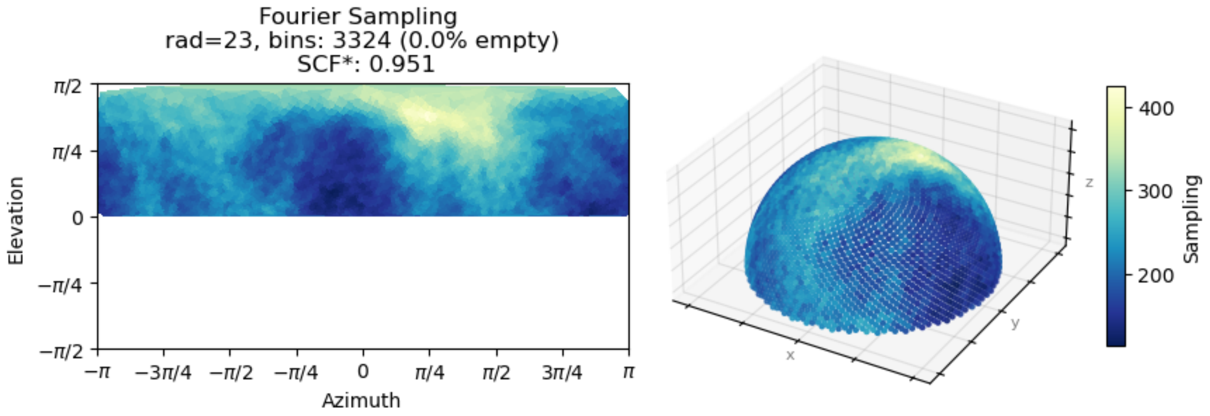


Conical FSC Area Ration (cFAR)[6]

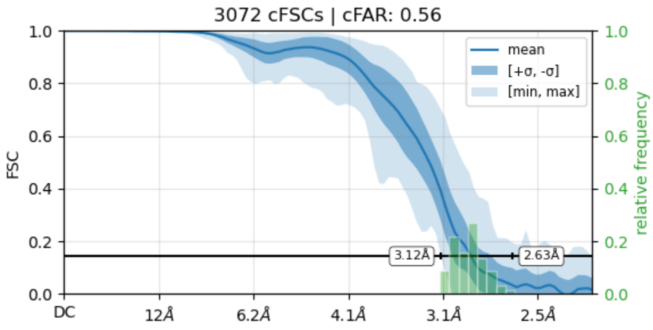


Gold Standard FSC

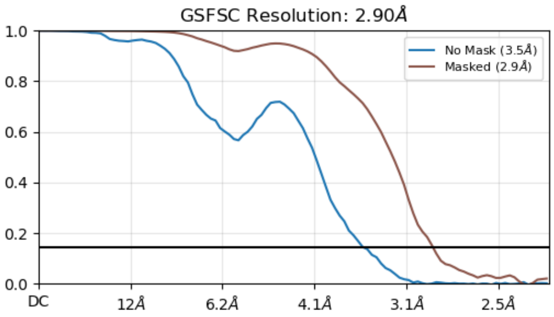


Posterior Precision Directional Distribution

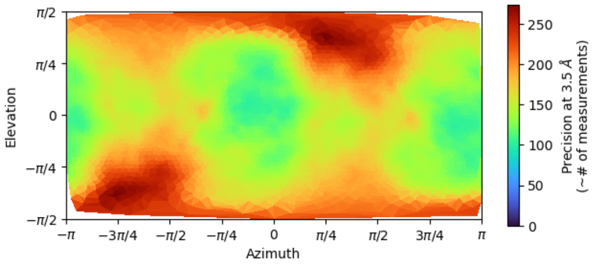


Viewing Direction Distribution

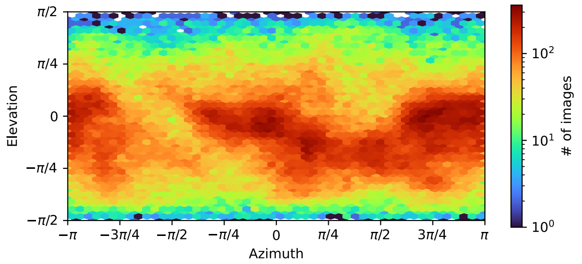


**Supplementary Fig. 14 | Orientation Diagnostics.** Output from Cryosparc 4.6.2. Input particles from final Local Refinement job, masked on the TM (overall resolution 2.9 Å). cFAR is based on 3DFSC.

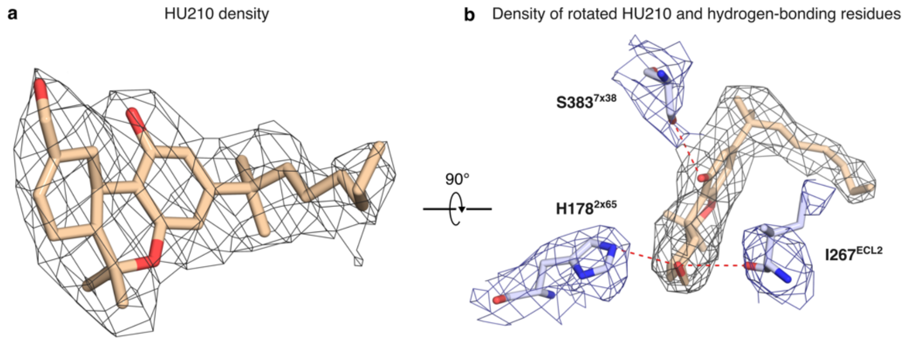


**Supplementary Fig. 15 | Cryo-EM density for the ligand HU210 and hydrogen-bonding residues in the $CB_1$ receptor. a,** Cryo-EM density (contoured at 0.6 σ) for HU210. **b,** Cryo-EM density (contoured at 0.6 σ) for HU210 and surrounding hydrogen-bonding CB1 residues.

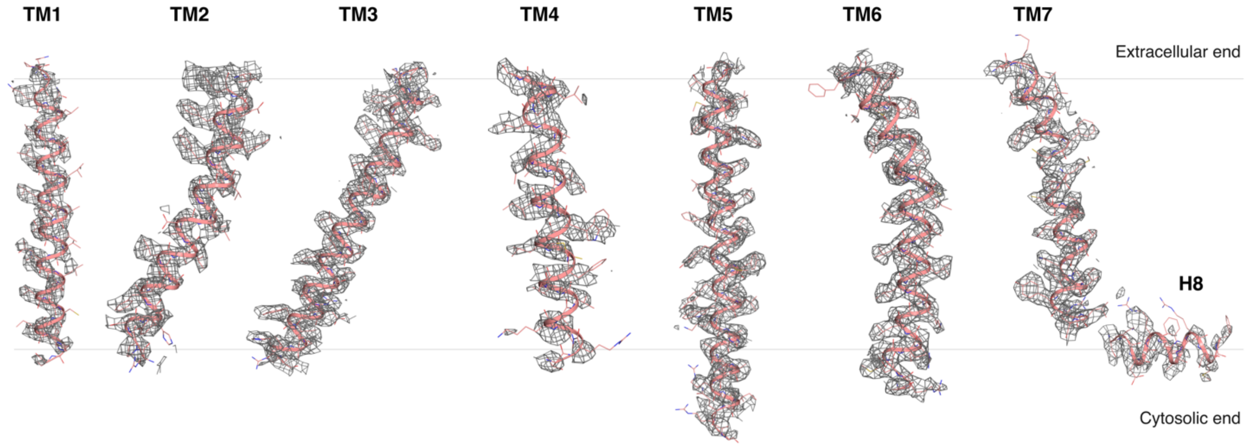


**Supplementary Fig. 16 | Cryo-EM density maps for each helix of the CB1 receptor.** Cryo-EM density (contoured at 0.7 $\sigma$) shown individually for each helix of the CB1 receptor – transmembrane helices 1-7 (TM1-7) and the C-terminal helix 8 (H8).

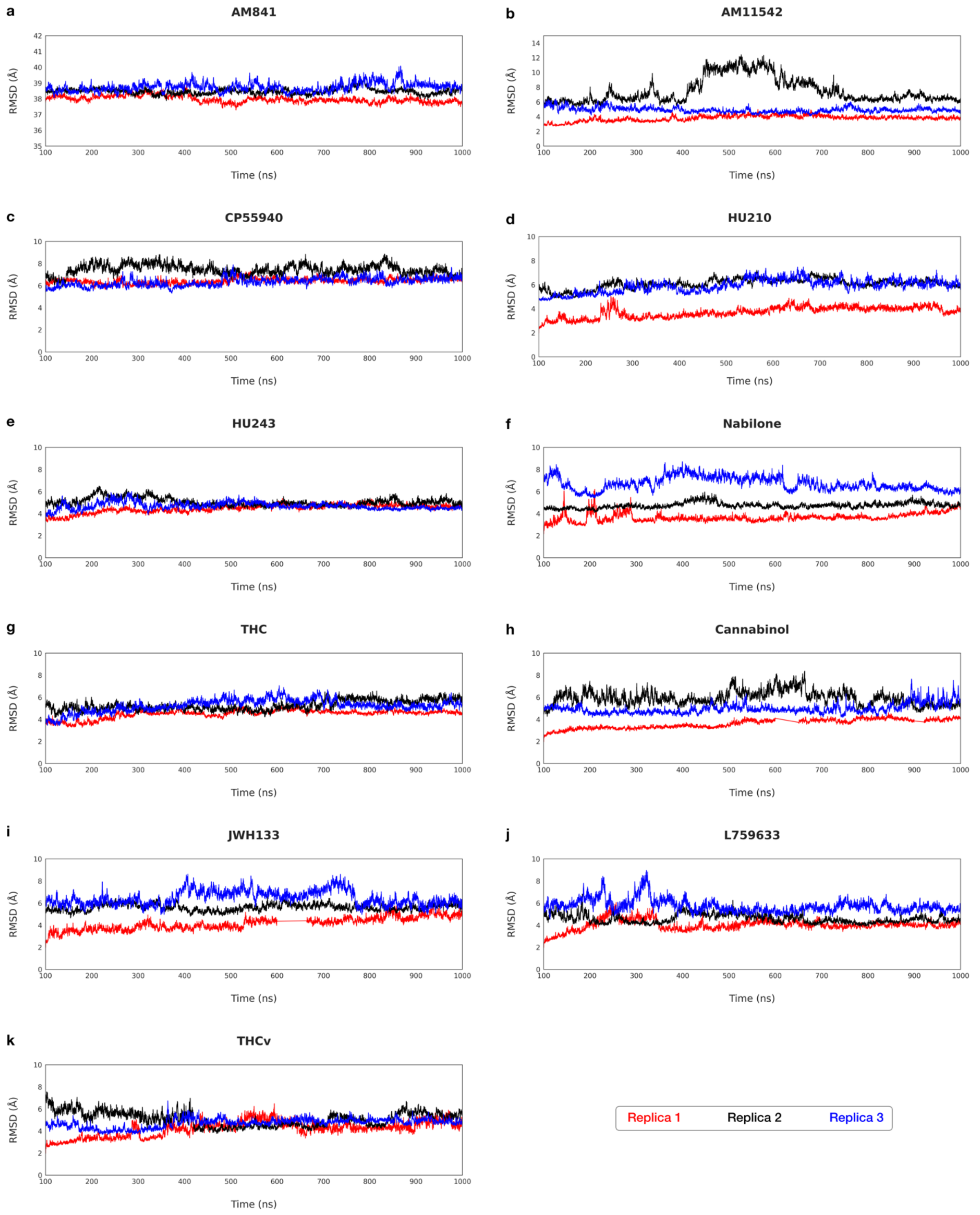


**Supplementary Fig. 17 | Root mean square deviation (RMSD).** Time-resolved RMSD calculated for CB1/Gi1 in complex with THC and its analogs in MD simulations over 1000 ns of trajectory. The first 100 ns have been omitted in the plots as part of system equilibration. Data within each plot correspond to individual replicates (Red: Replica 1, Black: Replica 2, Blue: Replica 3).

**Supplementary Fig. 18 | Heatmaps of residue-ligand contact frequencies.** Time-resolved graphical representation of contact frequencies for 29 interactions belonging to 25 residue-ligand pairs across 11 receptor-ligand complexes, as mentioned and ordered in Fig. 4b: **a:** AM841, **b:** AM11542, **c:** CP55940, **d:** HU210, **e:** HU243, **f:** Nabilone, **g:** THC, **h:** Cannabinol, **i:** JWH133, **j:** L759633, **k:** THCv. The interacting residues are grouped and colour-coded into the three categories – Tetrahydrocannabinol, Alkyl Branch and Alkyl Tail – as shown in Fig. 4. Four of the 29 contacts are hydrogen bond interactions and carry "Hb" as a label on top of the plot boxes. The rest of the plots correspond to hydrophobic or van der Waals interactions as indicated in Fig. 4. Data is derived from MD simulations for each receptor-ligand system, each comprising of 3 x 1000 ns trajectories.

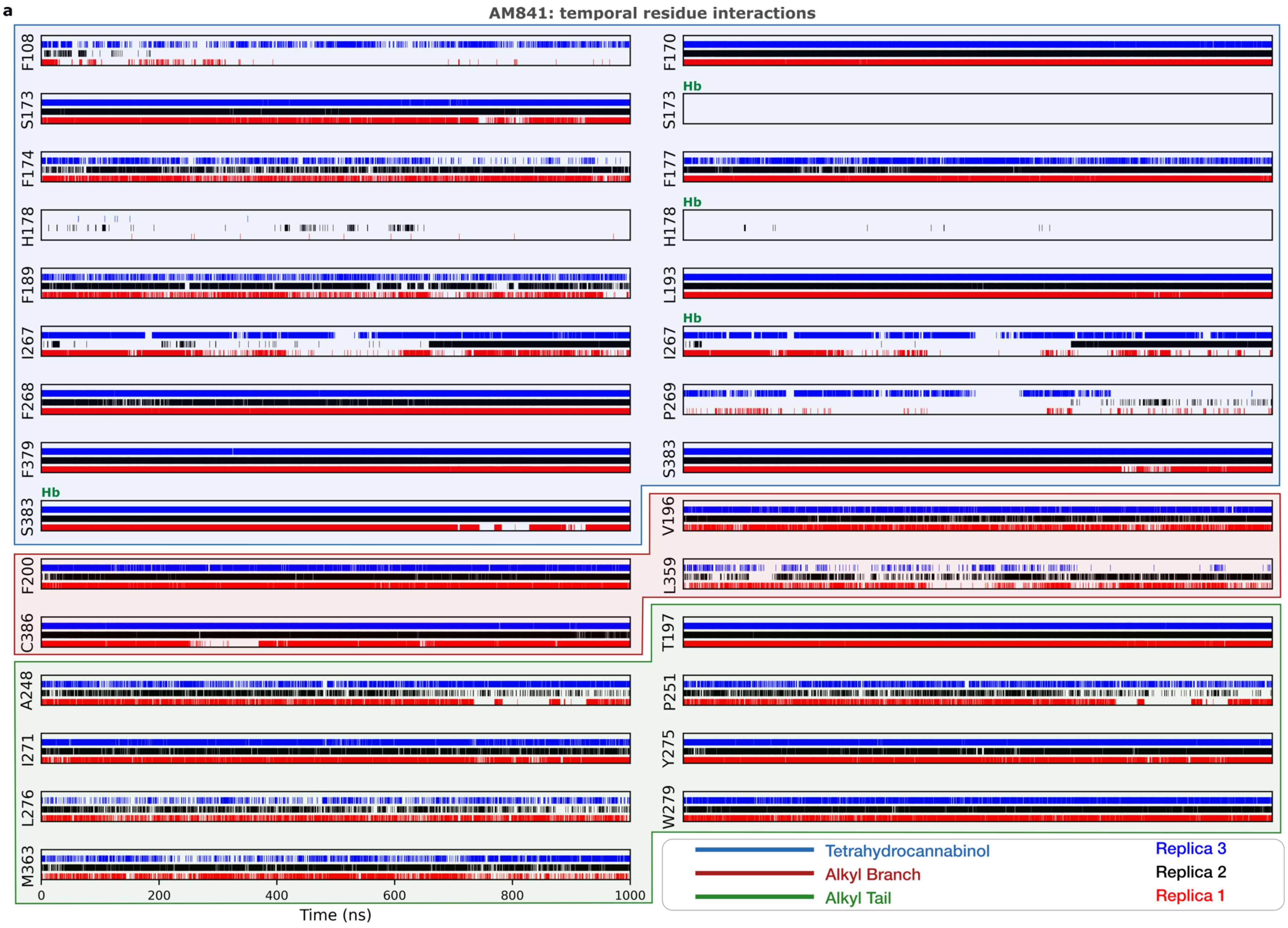
a
AM841: temporal residue interactions
F108
F170
S173
Hb
S173
F174
F177
H178
Hb
H178
F189
L193
I267
Hb
I267
F268
P269
F379
S383
Hb
S383
V196
F200
L359
C386
T197
A248
P251
I271
Y275
L276
W279
M363
0
200
400
600
800
1000
Time (ns)
Tetrahydrocannabinol
Alkyl Branch
Alkyl Tail
Replica 3
Replica 2
Replica 1

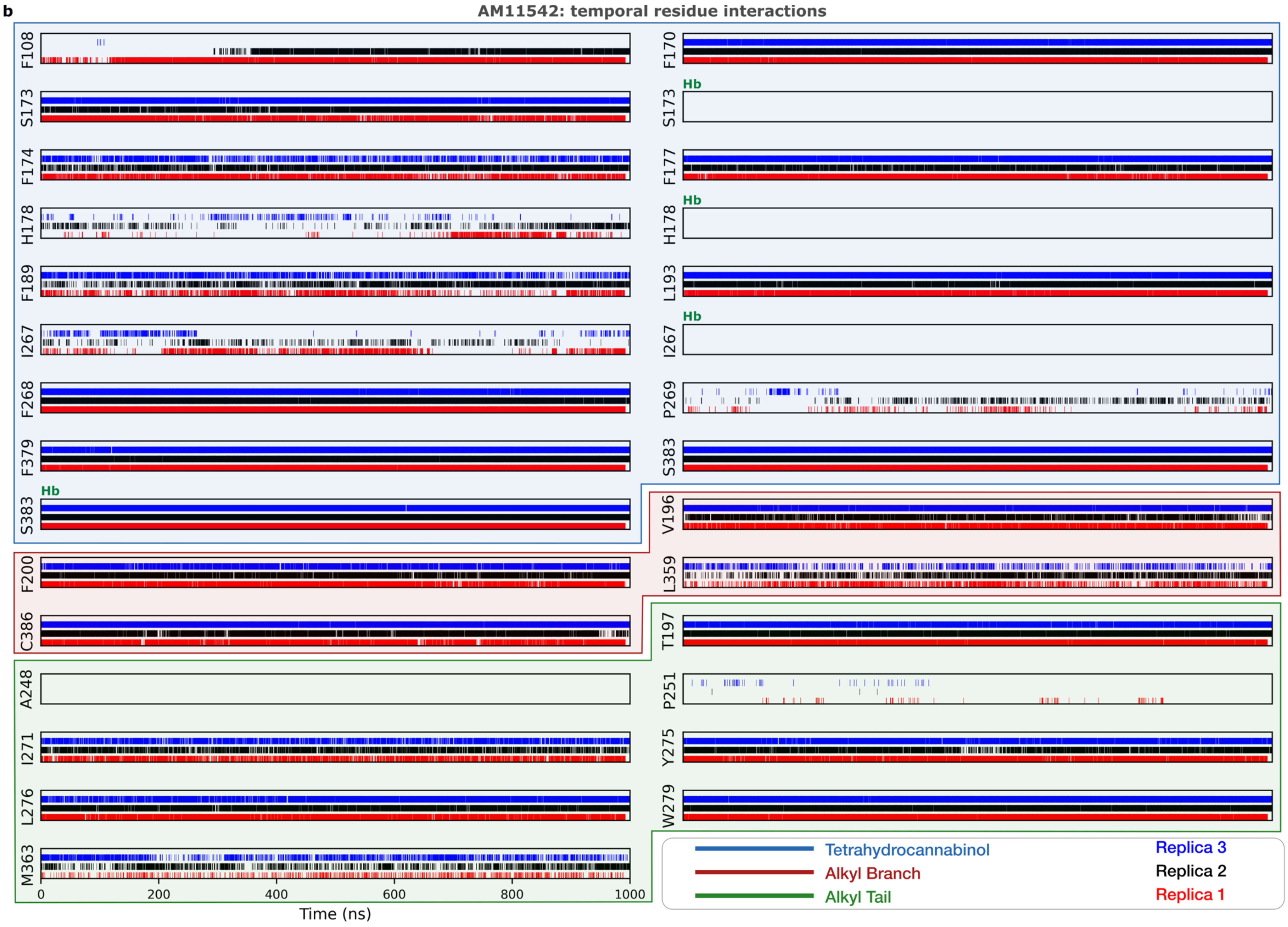

b
AM11542: temporal residue interactions
F108
S173
F174
H178
F189
I267
F268
F379
Hb
S383
F200
C386
A248
I271
L276
M363
F170
Hb
S173
F177
Hb
H178
L193
Hb
I267
P269
S383
V196
L359
T197
P251
Y275
W279
0
200
400
600
800
1000
Time (ns)
Tetrahydrocannabinol
Alkyl Branch
Alkyl Tail
Replica 3
Replica 2
Replica 1

c

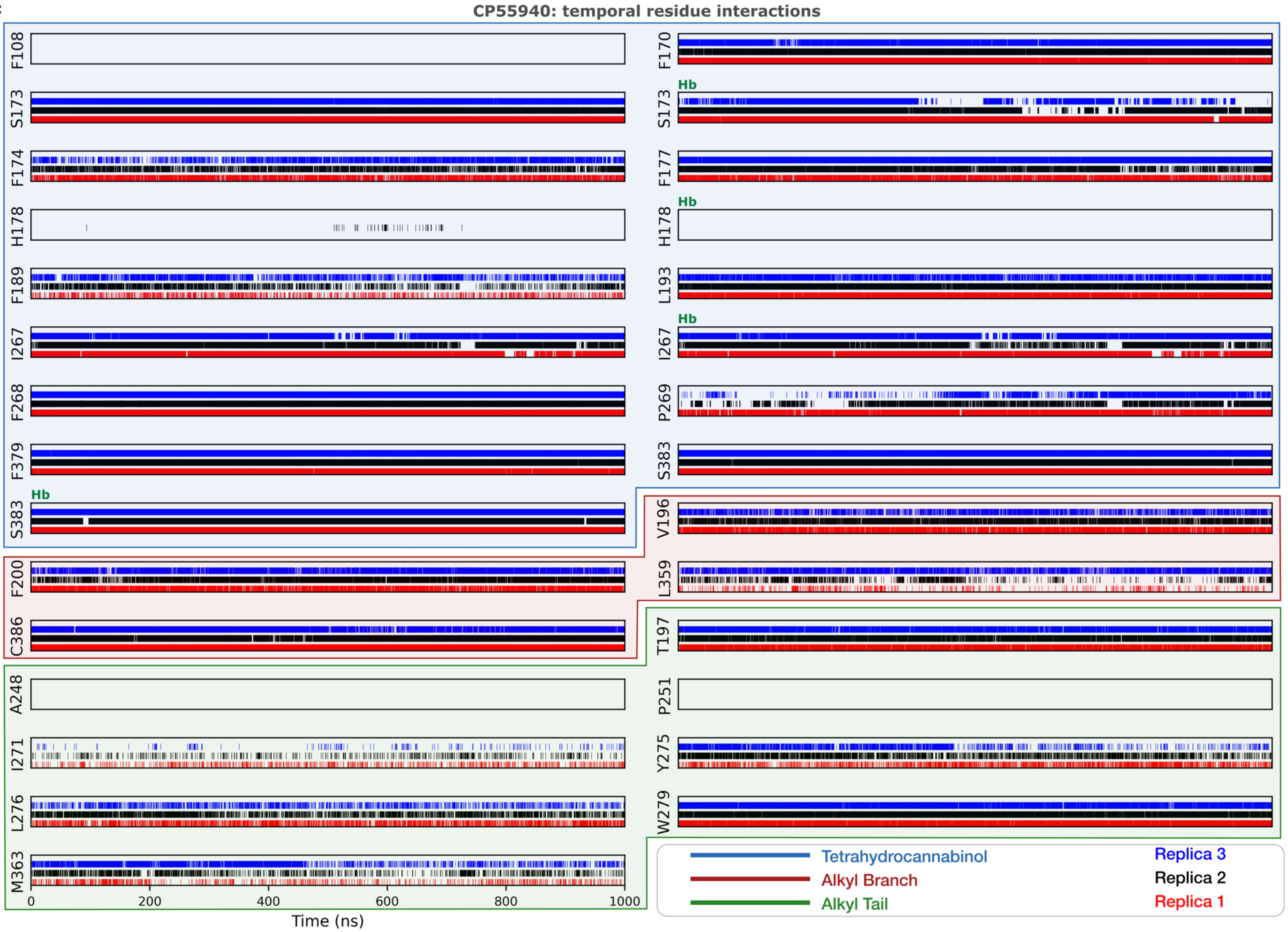
CP55940: temporal residue interactions
F108
S173
F174
H178
F189
I267
F268
F379
Hb
S383
F170
Hb
S173
F177
Hb
H178
L193
Hb
I267
P269
S383
V196
L359
F200
C386
T197
A248
P251
I271
Y275
L276
W279
M363
0
200
400
600
800
1000
Time (ns)
Tetrahydrocannabinol
Alkyl Branch
Alkyl Tail
Replica 3
Replica 2
Replica 1

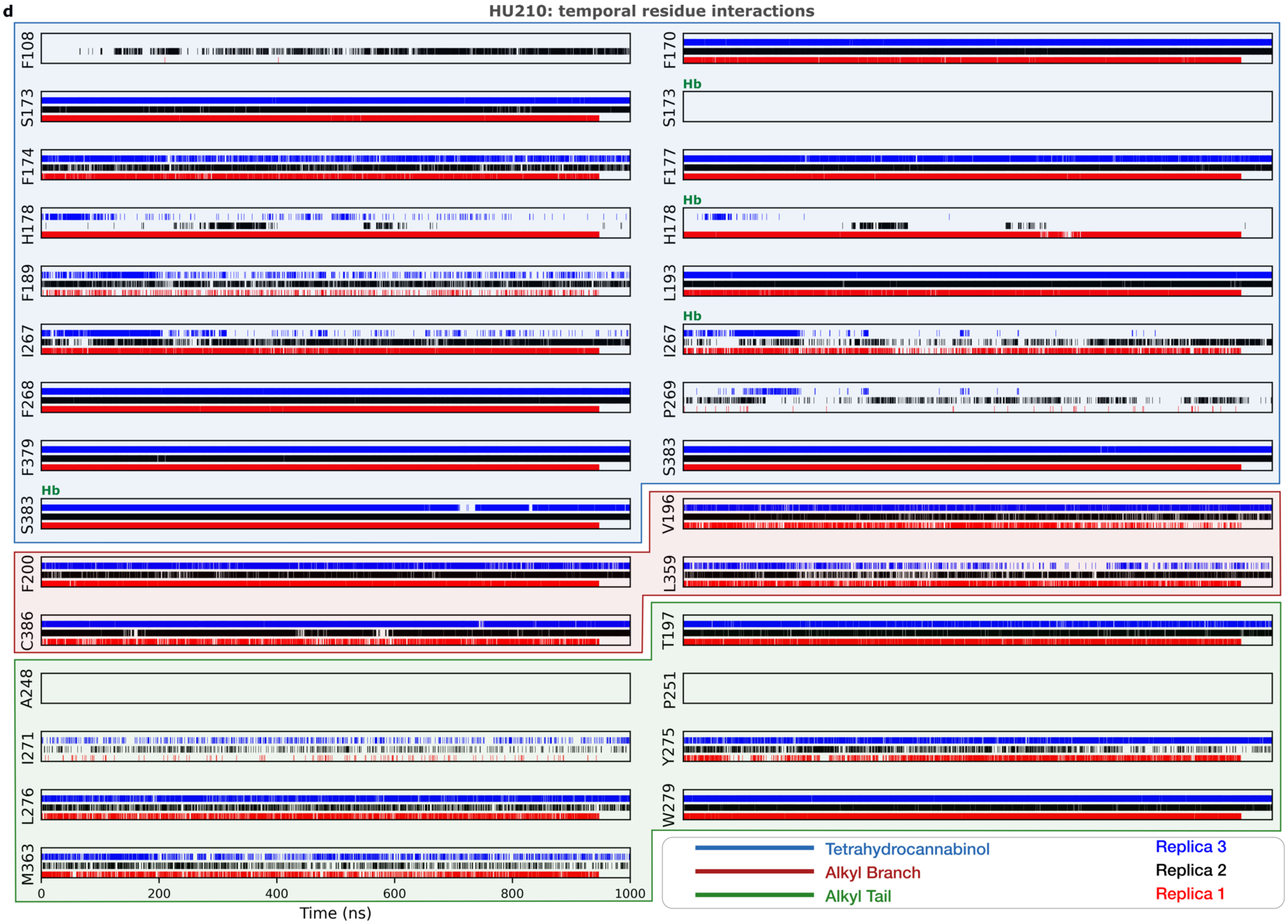
d
HU210: temporal residue interactions
F108
S173
F174
H178
F189
I267
F268
F379
Hb
S383
F200
C386
A248
I271
L276
M363
0
200
400
600
800
1000
Time (ns)
F170
Hb
S173
F177
Hb
H178
L193
Hb
I267
P269
S383
V196
L359
T197
P251
Y275
W279
Tetrahydrocannabinol
Alkyl Branch
Alkyl Tail
Replica 3
Replica 2
Replica 1

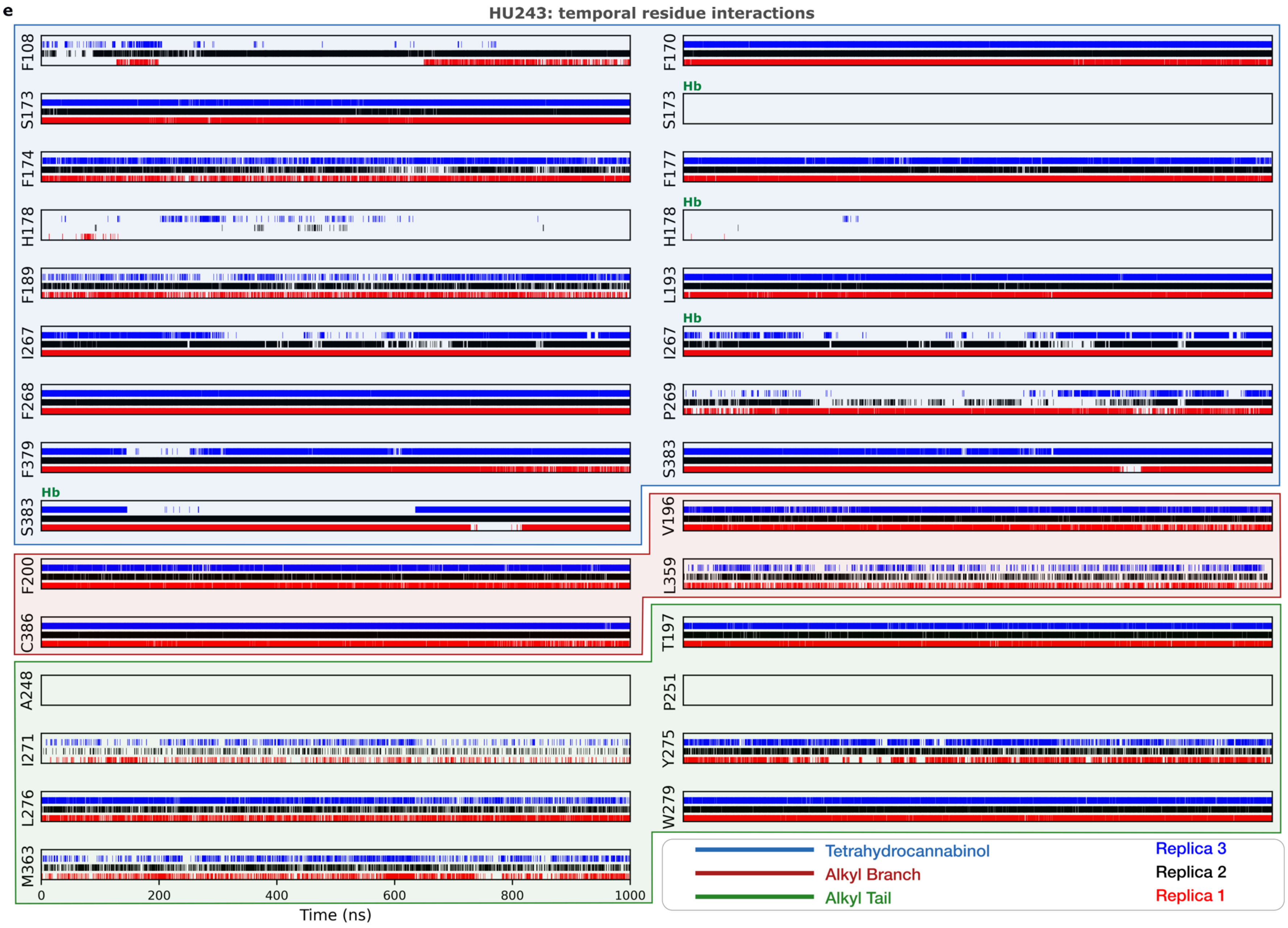
e
HU243: temporal residue interactions
F108
S173
F174
H178
F189
I267
F268
F379
Hb
S383
F170
Hb
S173
F177
Hb
H178
L193
Hb
I267
P269
S383
V196
L359
F200
C386
T197
A248
P251
I271
Y275
L276
W279
M363
0
200
400
600
800
1000
Time (ns)
Tetrahydrocannabinol
Alkyl Branch
Alkyl Tail
Replica 3
Replica 2
Replica 1

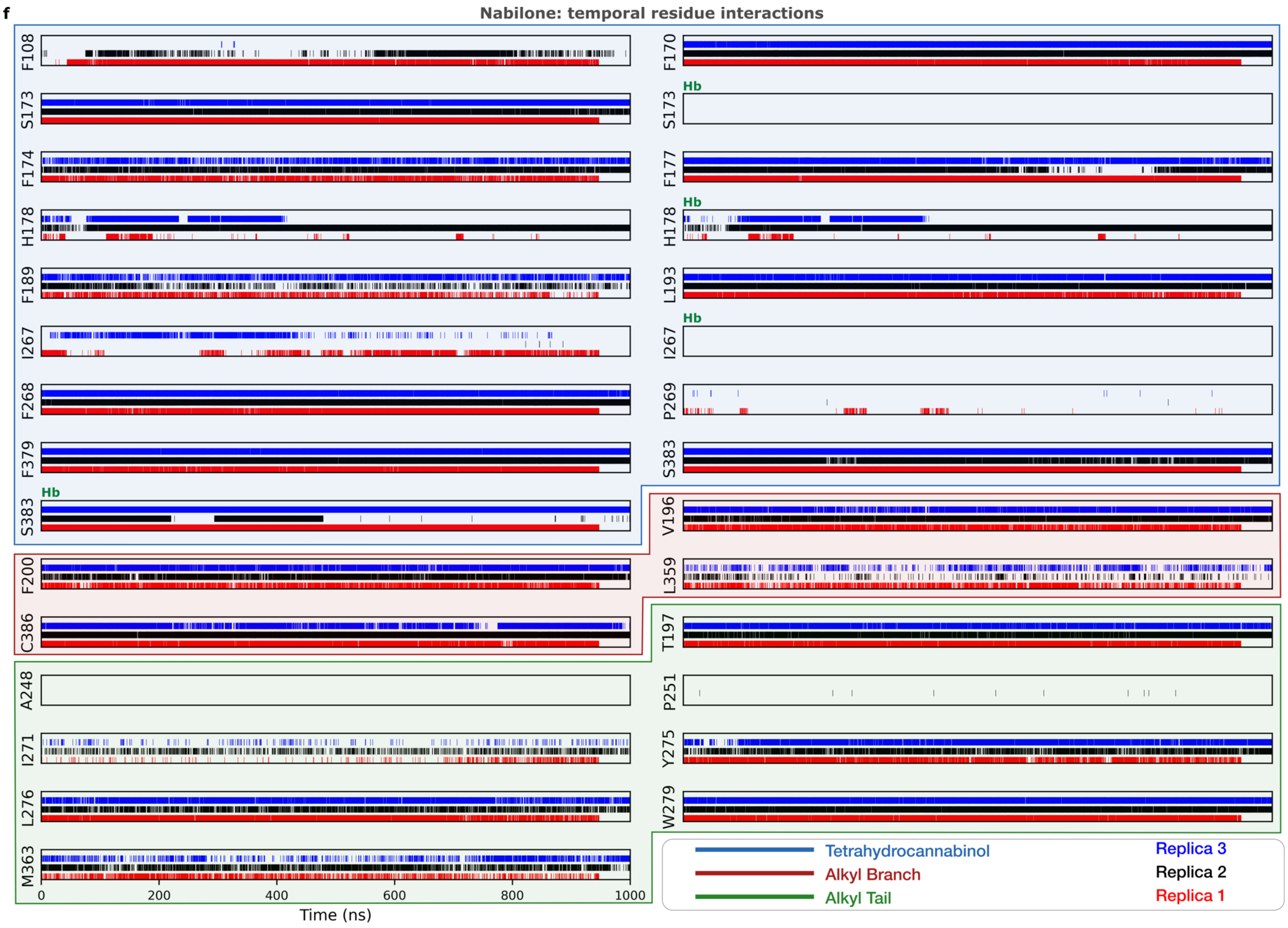
f
Nabilone: temporal residue interactions
F108
S173
F174
H178
F189
I267
F268
F379
Hb
S383
F200
C386
A248
I271
L276
M363
F170
Hb
S173
F177
Hb
H178
L193
Hb
I267
P269
S383
V196
L359
T197
P251
Y275
W279
0
200
400
600
800
1000
Time (ns)
Tetrahydrocannabinol
Alkyl Branch
Alkyl Tail
Replica 3
Replica 2
Replica 1

g

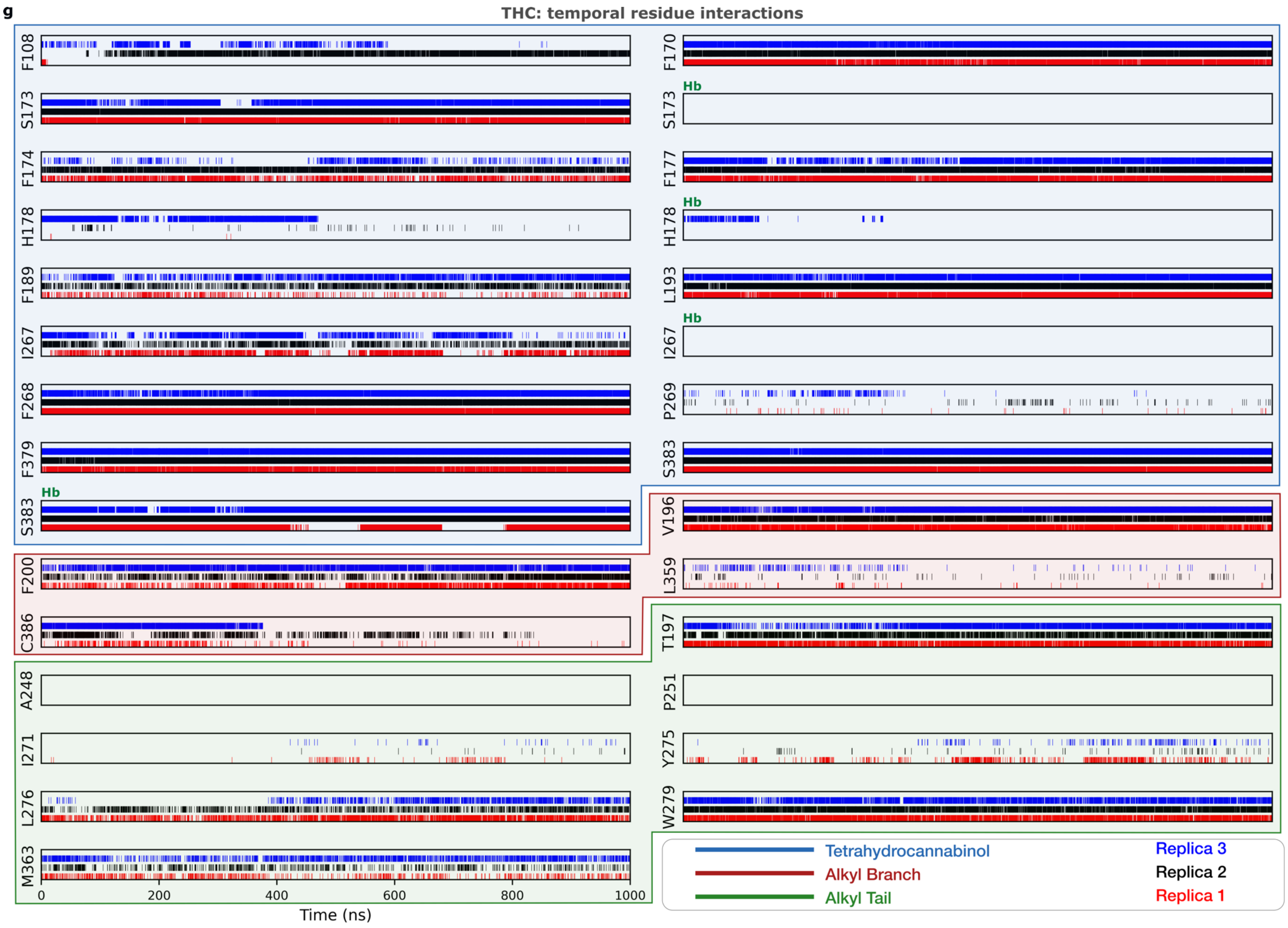
THC: temporal residue interactions
F108
S173
F174
H178
F189
I267
F268
F379
Hb
S383
F200
C386
A248
I271
L276
M363
0
200
400
600
800
1000
Time (ns)
F170
Hb
S173
F177
Hb
H178
L193
Hb
I267
P269
S383
V196
L359
T197
P251
Y275
W279
Tetrahydrocannabinol
Alkyl Branch
Alkyl Tail
Replica 3
Replica 2
Replica 1

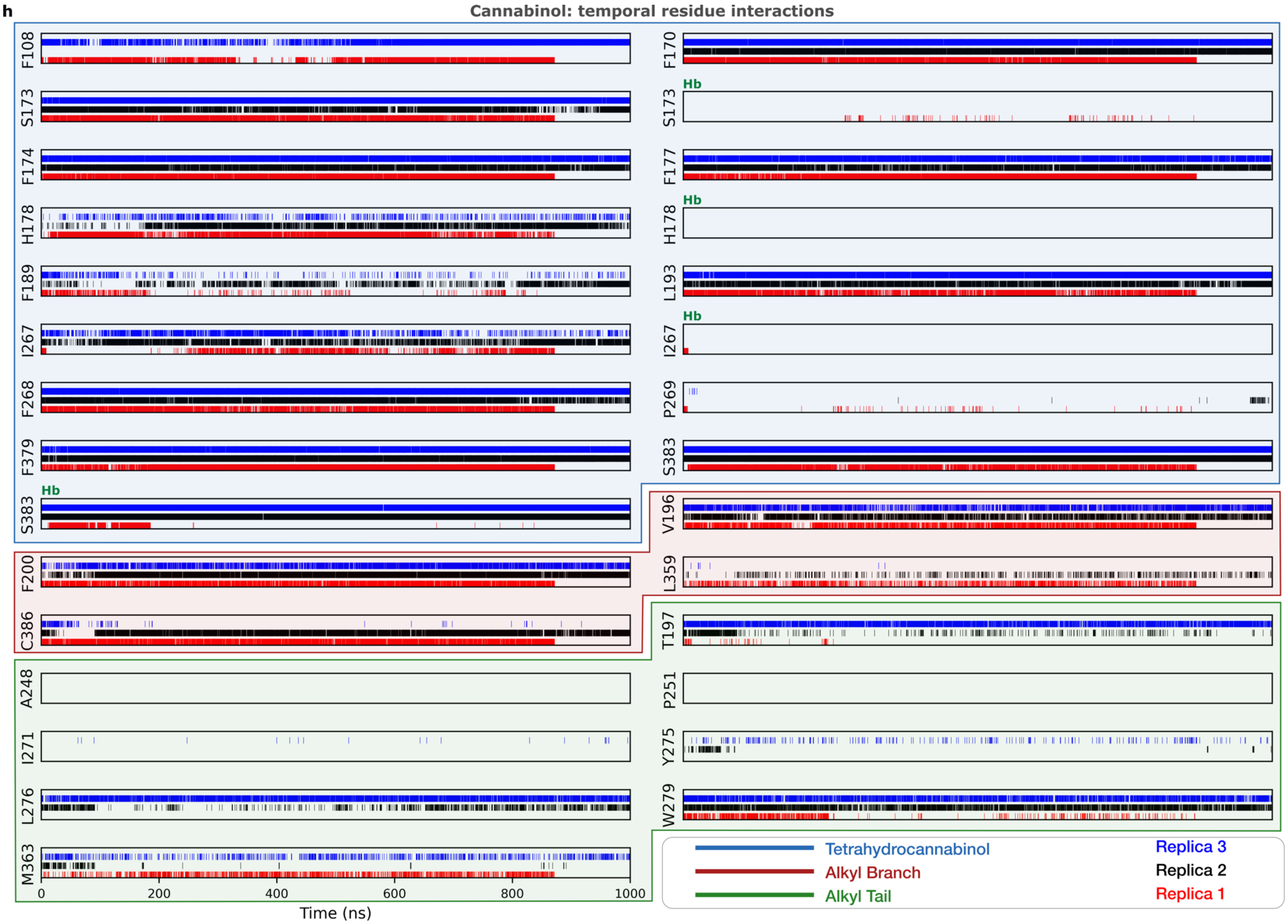

h
Cannabinol: temporal residue interactions
F108
S173
F174
H178
F189
I267
F268
F379
Hb
S383
F200
C386
A248
I271
L276
M363
0
200
400
600
800
1000
Time (ns)
F170
Hb
S173
F177
Hb
H178
L193
Hb
I267
P269
S383
V196
L359
T197
P251
Y275
W279
Tetrahydrocannabinol
Alkyl Branch
Alkyl Tail
Replica 3
Replica 2
Replica 1

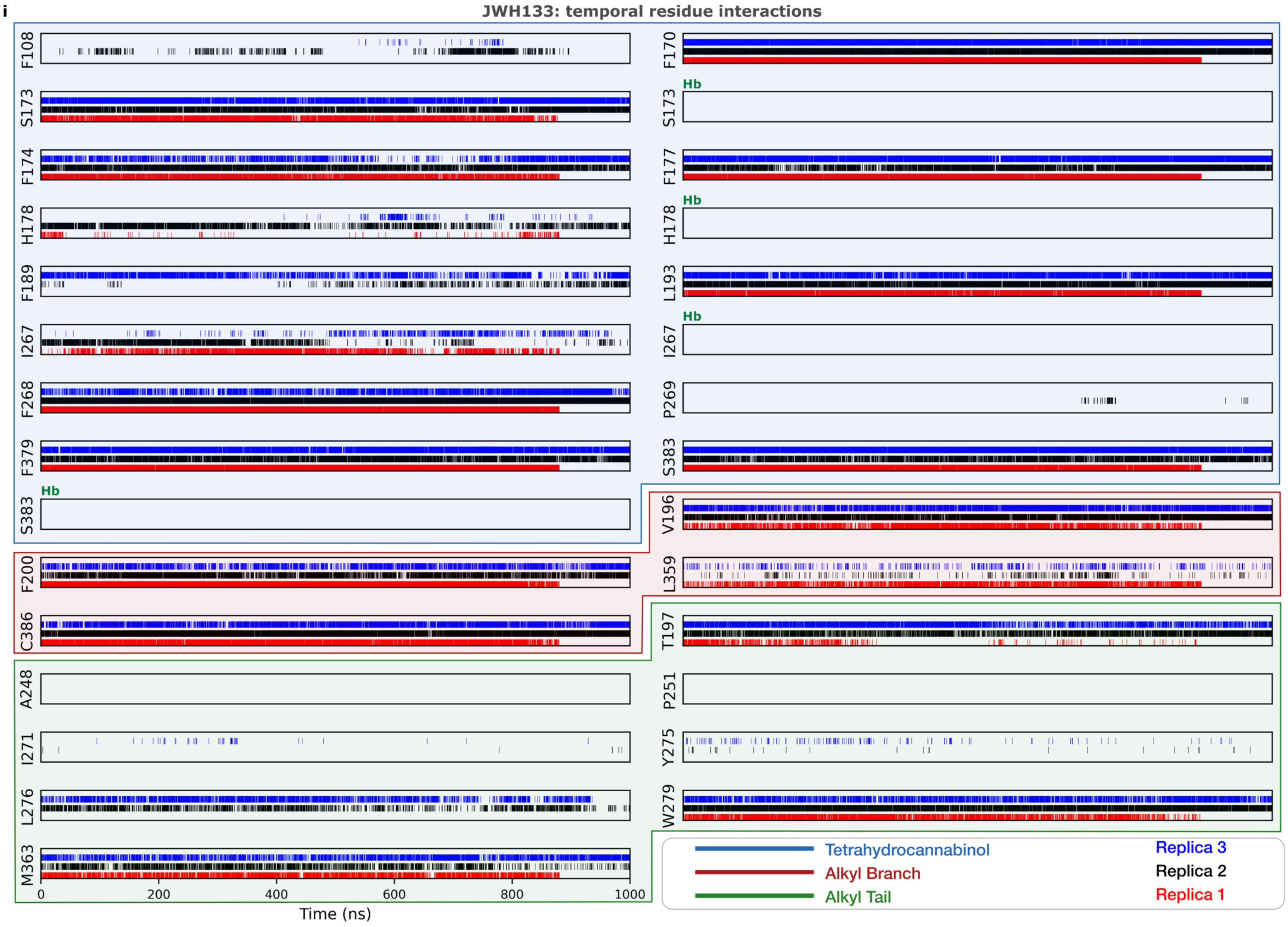
i
JWH133: temporal residue interactions
F108
S173
F174
H178
F189
I267
F268
F379
Hb
S383
F200
C386
A248
I271
L276
M363
0
200
400
600
800
1000
Time (ns)
F170
Hb
S173
F177
Hb
H178
L193
Hb
I267
P269
S383
V196
L359
T197
P251
Y275
W279
Tetrahydrocannabinol
Alkyl Branch
Alkyl Tail
Replica 3
Replica 2
Replica 1

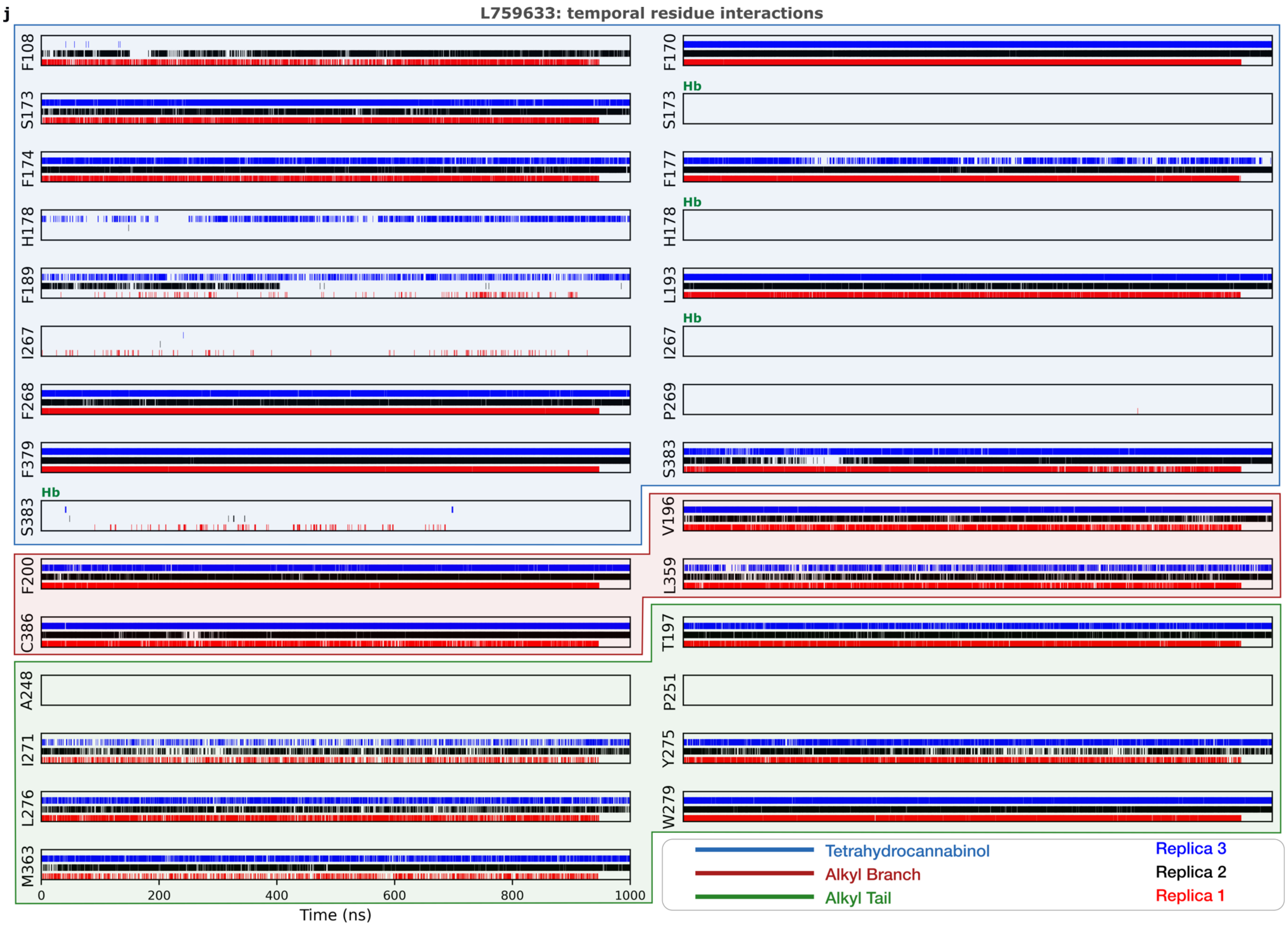
j
L759633: temporal residue interactions
F108
S173
F174
H178
F189
I267
F268
F379
Hb
S383
F170
Hb
S173
F177
Hb
H178
L193
Hb
I267
P269
S383
V196
L359
F200
C386
T197
A248
P251
I271
Y275
L276
W279
M363
0
200
400
600
800
1000
Time (ns)
Tetrahydrocannabinol
Alkyl Branch
Alkyl Tail
Replica 3
Replica 2
Replica 1

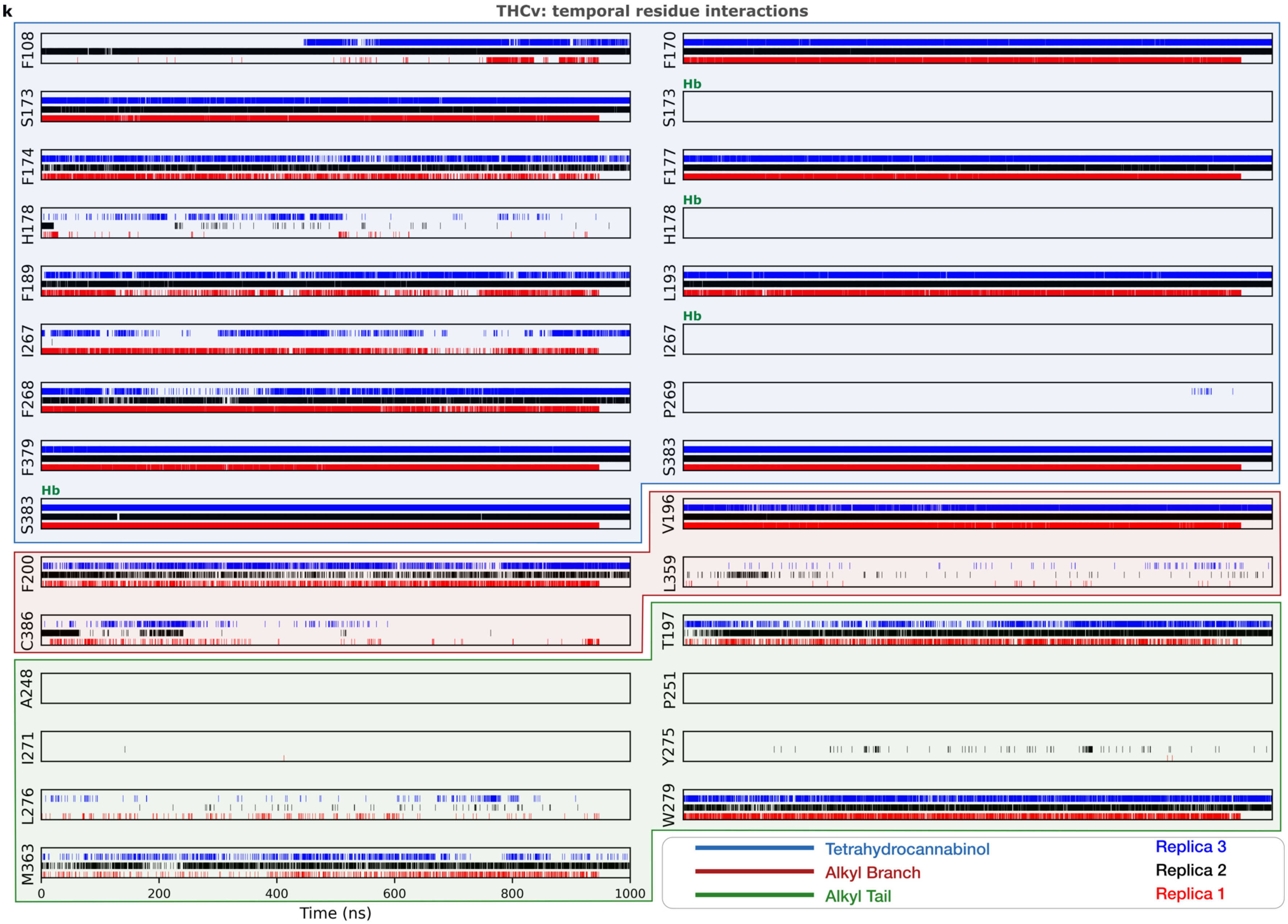

k
THCv: temporal residue interactions
F108
S173
F174
H178
F189
I267
F268
F379
Hb
S383
F200
C386
A248
I271
L276
M363
0
200
400
600
800
1000
Time (ns)
F170
Hb
S173
F177
Hb
H178
L193
Hb
I267
P269
S383
V196
L359
T197
P251
Y275
W279
Tetrahydrocannabinol
Alkyl Branch
Alkyl Tail
Replica 3
Replica 2
Replica 1

**Supplementary Table 1 | Active state structures of $CB_1$ and the AM12033/$CB_2$ complex[7].** *Tanimoto coefficients.

| PDB ID | Ref | Res (Å) | Method | Ligand | THC-analog | *Ligand similarity to $\Delta^9$-THC | *Ligand similarity to HU210 | G prot-ein | Pref-erred chain | RMSD to 9ERX (7TM, Å) | Receptor residues (%) | Receptor backbones (complete) | Receptor sidechains (complete) | Receptor mutations |
|---|---|---|---|---|---|---|---|---|---|---|---|---|---|---|
| **8GHV** | 8 | 2.8 | Cryo-EM | AMG315 | No | 0.11 | 0.12 | $G_{i1}$ | D | 0.82 | 59 | 280 | 217 | - |
| **6N4B** | 9 | 3.0 | Cryo-EM | MDMB-Fubinaca (FUB) | No | 0.33 | 0.34 | $G_{i1}$ | R | 0.90 | 58 | 276 | 205 | - |
| **9ERX** | - | 2.9 | Cryo-EM | HU210 | Yes | 0.82 | 1.00 | $G_{i1}$ | R | - | 62 | 294 | 294 | - |
| **6KPG** | 7 | 3.0 | Cryo-EM | AM841 | Yes | 0.77 | 0.82 | $G_{i1}$ | R | 0.81 | 60 | 285 | 270 | $E273^{5x37}$ $T283^{5x47}$ $R340^{6x32}$ |
| **7WV9** | 10 | 3.4 | Cryo-EM | CP55940 + PAM | Yes | 0.46 | 0.49 | $G_{i2}$ | R | 0.94 | 61 | 290 | 287 | - |
| **5XRA** | 11 | 2.8 | X-ray | AM11542 | Yes | 0.84 | 0.96 | - | A | 1.14 | 60 | 280 | 276 | $T210^{3x46}$ $E273^{5x37}$ $T283^{5x47}$ $R340^{6x32}$ |
| **5XR8** | 11 | 3.0 | X-ray | AM841 | Yes | 0.77 | 0.82 | - | A | 1.28 | 60 | 284 | 277 | $T210^{3x46}$ $E273^{5x37}$ $T283^{5x47}$ $R340^{6x32}$ |
| **7V3Z** | 12 | 3.3 | X-ray | CP55940 | Yes | 0.46 | 0.49 | - | A | 1.18 | 60 | 284 | 277 | $H154^{2x41}$ $T210^{3x46}$ $E273^{5x37}$ $T283^{5x47}$ $R340^{6x32}$ |
| **6KPF** | 7 | 2.9 | Cryo-EM | AM12033 | Yes | 0.77 | 0.83 | $G_{i1}$ | R | 1.09 | 62 | 291 | 290 | - |

**Supplementary Table 2 | Pharmacological parameters of studied $CB_1$ agonists.**

| Ligand | $mG_i$ $pEC_{50}$ | SEM | $mG_i$ $E_{max}$ | SEM | $mG_i$ slope | SEM | n | Arr $pEC_{50}$ | SEM | Arr $E_{max}$ | SEM | Arr slope | SEM | n |
|---|---|---|---|---|---|---|---|---|---|---|---|---|---|---|
| 2AG | 6.00 | 0.22 | 100.0 | 4.9 | 0.59 | 0.04 | 6 | 5.28 | 0.18 | 100.0 | 15.4 | 0.63 | 0.03 | 6 |
| AEA | 6.35 | 0.10 | 57.9 | 1.6 | 0.81 | 0.06 | 9 | 5.85 | 0.23 | 30.5 | 4.0 | 1.15 | 0.25 | 9 |
| AM11542 | 8.47 | 0.07 | 54.6 | 1.9 | 1.09 | 0.06 | 6 | 8.16 | 0.07 | 31.7 | 3.5 | 1.13 | 0.15 | 6 |
| AM841 | 8.47 | 0.06 | 61.1 | 2.4 | 1.16 | 0.06 | 6 | 8.08 | 0.11 | 39.3 | 3.4 | 0.80 | 0.07 | 5 |
| Cannabinol | 6.89 | 0.07 | 15.0 | 1.4 | 1.37 | 0.22 | 6 | | | No response | | | | |
| CP55940 | 8.42 | 0.05 | 69.0 | 1.6 | 0.86 | 0.04 | 17 | 7.97 | 0.08 | 41.1 | 1.8 | 1.00 | 0.13 | 12 |
| HU210 | 8.62 | 0.07 | 66.5 | 2.3 | 1.02 | 0.13 | 6 | 8.10 | 0.12 | 40.2 | 4.7 | 0.91 | 0.11 | 6 |
| HU243 | 8.69 | 0.09 | 74.1 | 2.0 | 1.04 | 0.10 | 6 | 8.11 | 0.15 | 58.5 | 4.4 | 0.76 | 0.08 | 6 |
| JWH133 | 5.94 | 0.10 | 23.7 | 1.5 | 1.40 | 0.23 | 5 | 6.05 | 0.42 | 9.1 | 3.1 | 4.95 | 4.38 | 3 |
| L759633 | 6.38 | 0.26 | 21.5 | 4.4 | 0.89 | 0.24 | 3 | | | No fit | | | | |
| Nabilone | 7.86 | 0.11 | 75.8 | 3.9 | 0.78 | 0.04 | 6 | 7.11 | 0.16 | 58.8 | 4.8 | 0.96 | 0.34 | 4 |
| THC | 8.29 | 0.10 | 24.0 | 1.4 | 0.93 | 0.11 | 12 | 7.90 | 0.24 | 14.1 | 2.9 | 0.63 | 0.15 | 6 |
| THCv | 6.70 | 0.15 | -13.3 | 1.3 | 1.01 | 0.10 | 6 | | | No fit | | | | |
| WIN55212-2* | 6.70 | 0.04 | 85.8 | 3.0 | 0.73 | 0.04 | 6 | | | No response | | | | |

*WIN55212-2 is not a THC analog but is included here because its $G_{i1}$ recruitment data is part of the Supplementary Fig. 5-6.

**Supplementary Table 3 | Cryo-EM data collection, refinement, and validation statistics.**

| | (EMDB-19929) (PDB 9ERX) |
|---|---|
| **Data collection and processing** | |
| Magnification | 130,000 |
| Voltage (kV) | 300 |
| Electron exposure (e–/Å²) | 62 |
| Defocus range (μm) | 600-1800 |
| Pixel size (Å) | .6435 |
| Symmetry imposed | none |
| Initial particle images (no.) | 603,000 |
| Final particle images (no.) | 187,000 |
| Map resolution (Å) | 2.9 |
| FSC threshold | 0.143 |
| Map resolution range (Å) | 1.54-4.19 |
| **Refinement** | |
| Initial model used (PDB code) | 6KPG, 6CRK |
| Model resolution (Å) | 3.0 |
| FSC threshold | 0.143 |
| Model resolution range (Å) | 1.29-4.38 |
| Map sharpening B factor (Å²) | 0 |
| Model composition | |
| Non-hydrogen atoms | 9021 |
| Protein residues | 1150 |
| Ligands | 1 |
| B factors (Å²) | |
| Protein | 55.3 |
| Ligand | 45.2 |
| R.m.s. deviations | |
| Bond lengths (Å) | 0.004 |
| Bond angles (°) | 0.746 |
| **Validation** | |
| MolProbity score | 1.6 |
| Clashscore | 5 |
| Poor rotamers (%) | |
| Ramachandran plot | |
| Favored (%) | 95 |
| Allowed (%) | 5 |
| Disallowed (%) | 0 |

**Supplementary Table 4 | Slow or fast dissociation of THC analogs and AEA to $CB_1$ in this study and in literature.** Data from our study is from HEK293-TR cells stably expressing $CB_1$-NlucC and NES-venus-$mG_i$. $mG_i$ responses are shown as mean ± S.E.M. from at least three independent experiments. N-value is 6 for each of the individual ligand dissociation curves except for THC (n=5). The underlying graph, including controls, for each ligand is provided in Supplementary Figure 8.

| Ligand | Published qualitative description | Our qualitative description | $k_{off}$ ($min^{-1}$) | Half-life (min) |
|---|---|---|---|---|
| THC | - | ”Fast” (BRET) | 0.5718 ± 0.0560 | 1.2 ± 0.1 |
| AEA | - | “Fast” (BRET) | 0.5976 ± 0.0231 | 1.2 ± 0.0 |
| AM11245 | “Tight” (Washout)[13] | - | - | - |
| AM11542 | “Wash-resistant” (Radioligand binding assay)[11] | “Very slow” (BRET) | 0.0115 ± 0.0003 | 60.6 ± 1.4 |
| AM841 | “Wash-resistant” (Radioligand binding assay)[11] “Tight” (Washout)[13] “Irreversible” (Washout)[14] “Covalent” (Washout + mass spectrometry)[15] | “Very slow” (BRET) | 0.0140 ± 0.0002 | 49.6 ± 0.7 |
| CP55940 | - | “Fast” (BRET) | 0.1681 ± 0.0041 | 4.1 ± 0.1 |
| HU210 | “Pseudo-irreversible” (Drug discrimination, $ED_{50}$)[16] | “Very slow” (BRET) | 0.0224 ± 0.0002 | 31.0 ± 0.2 |
| HU243 | “Incapable of irreversible association” (Washout)[14] | “Very slow” (BRET) | 0.0123 ± 0.0002 | 56.5 ± 0.8 |

## References


1 Miljuš, T. *et al.* Diverse chemotypes drive biased signaling by cannabinoid receptors. *bioRxiv*, 2020.2011.2009.375162, doi:10.1101/2020.11.09.375162 (2020).

2 Khajehali, E. *et al.* Biased Agonism and Biased Allosteric Modulation at the CB1 Cannabinoid Receptor. *Molecular pharmacology* **88**, 368-379, doi:10.1124/mol.115.099192 (2015).

3 Zhu, X., Finlay, D. B., Glass, M. & Duffull, S. B. Evaluation of the profiles of CB1 cannabinoid receptor signalling bias using joint kinetic modelling. *Br. J. Pharmacol.* **177**, 3449-3463, doi:https://doi.org/10.1111/bph.15066 (2020).

4 Cardone, G., Heymann, J. B. & Steven, A. C. One number does not fit all: mapping local variations in resolution in cryo-EM reconstructions. *J. Struct. Biol.* **184**, 226-236, doi:10.1016/j.jsb.2013.08.002 (2013).

5 Baldwin, P. R. & Lyumkis, D. Tools for visualizing and analyzing Fourier space sampling in Cryo-EM. *Prog Biophys Mol Biol* **160**, 53-65, doi:10.1016/j.pbiomolbio.2020.06.003 (2021).

6 Tan, Y. Z. *et al.* Addressing preferred specimen orientation in single-particle cryo-EM through tilting. *Nat. Methods* **14**, 793-796, doi:10.1038/nmeth.4347 (2017).

7 Hua, T. *et al.* Activation and Signaling Mechanism Revealed by Cannabinoid Receptor-G(i) Complex Structures. *Cell* **180**, 655-665 e618, doi:10.1016/j.cell.2020.01.008 (2020).

8 Krishna Kumar, K. *et al.* Structural basis for activation of CB1 by an endocannabinoid analog. *Nat Commun* **14**, 2672, doi:10.1038/s41467-023-37864-4 (2023).

9 Krishna Kumar, K. *et al.* Structure of a Signaling Cannabinoid Receptor 1-G Protein Complex. *Cell* **176**, 448-458 e412, doi:10.1016/j.cell.2018.11.040 (2019).

10 Yang, X. *et al.* Molecular mechanism of allosteric modulation for the cannabinoid receptor CB1. *Nat. Chem. Biol.* **18**, 831-840, doi:10.1038/s41589-022-01038-y (2022).

11 Hua, T. *et al.* Crystal structures of agonist-bound human cannabinoid receptor CB(1). *Nature* **547**, 468-471, doi:10.1038/nature23272 (2017).

12 Wang, X. *et al.* A Genetically Encoded F-19 NMR Probe Reveals the Allosteric Modulation Mechanism of Cannabinoid Receptor 1. *J. Am. Chem. Soc.* **143**, 16320-16325, doi:10.1021/jacs.1c06847 (2021).

13 Jiang, S. *et al.* Novel Functionalized Cannabinoid Receptor Probes: Development of Exceptionally Potent Agonists. *Journal of medicinal chemistry* **64**, 3870-3884, doi:10.1021/acs.jmedchem.0c02053 (2021).

14 Picone, R. P. *et al.* (-)-7'-Isothiocyanato-11-hydroxy-1',1'-dimethylheptylhexahydrocannabinol (AM841), a high-affinity electrophilic ligand, interacts covalently with a cysteine in helix six and activates the CB1 cannabinoid receptor. *Molecular pharmacology* **68**, 1623-1635, doi:10.1124/mol.105.014407 (2005).

15 Szymanski, D. W. *et al.* Mass spectrometry-based proteomics of human cannabinoid receptor 2: covalent cysteine 6.47(257)-ligand interaction affording megagonist receptor activation. *J. Proteome Res.* **10**, 4789-4798, doi:10.1021/pr2005583 (2011).

16 Hruba, L. & McMahon, L. R. The cannabinoid agonist HU-210: Pseudo-irreversible discriminative stimulus effects in rhesus monkeys. *Eur. J. Pharmacol.* **727**, 35-42, doi:https://doi.org/10.1016/j.ejphar.2014.01.041 (2014).
