# Supplementary material for: Structural basis of THC analog activity at the Cannabinoid 1 receptor
Source: Nat Commun. 2025 Jan 8;16:486. doi: 10.1038/s41467-024-55808-4 (PMC11711184; doi:10.1038/s41467-024-55808-4)
Supplement: Supplementary file 2 — Description of Additional Supplementary Files [file 41467_2024_55808_MOESM2_ESM.pdf]

File name: Supplementary Data 1

Description: Microsoft Excel file with ligand bias calculations.
