# Supplementary material for: Structural basis of THC analog activity at the Cannabinoid 1 receptor
Source: Nat Commun. 2025 Jan 8;16:486. doi: 10.1038/s41467-024-55808-4 (PMC11711184; doi:10.1038/s41467-024-55808-4)
Supplement: Supplementary file 4 — Reporting Summary [file 41467_2024_55808_MOESM4_ESM.pdf]

nature portfolio

Corresponding author(s): David Gloriam

Last updated by author(s): Dec 17, 2024

Reporting Summary

Nature Portfolio wishes to improve the reproducibility of the work that we publish. This form provides structure for consistency and transparency in reporting. For further information on Nature Portfolio policies, see our Editorial Policies and the Editorial Policy Checklist.

Statistics

For all statistical analyses, confirm that the following items are present in the figure legend, table legend, main text, or Methods section.

n/a	Confirmed	
☐	☒	The exact sample size (*n*) for each experimental group/condition, given as a discrete number and unit of measurement
☐	☒	A statement on whether measurements were taken from distinct samples or whether the same sample was measured repeatedly
☒	☐	The statistical test(s) used AND whether they are one- or two-sided<br>*Only common tests should be described solely by name; describe more complex techniques in the Methods section.*
☒	☐	A description of all covariates tested
☐	☒	A description of any assumptions or corrections, such as tests of normality and adjustment for multiple comparisons
☐	☒	A full description of the statistical parameters including central tendency (e.g. means) or other basic estimates (e.g. regression coefficient) AND variation (e.g. standard deviation) or associated estimates of uncertainty (e.g. confidence intervals)
☒	☐	For null hypothesis testing, the test statistic (e.g. *F*, *t*, *r*) with confidence intervals, effect sizes, degrees of freedom and *P* value noted<br>*Give P values as exact values whenever suitable.*
☒	☐	For Bayesian analysis, information on the choice of priors and Markov chain Monte Carlo settings
☒	☐	For hierarchical and complex designs, identification of the appropriate level for tests and full reporting of outcomes
☒	☐	Estimates of effect sizes (e.g. Cohen's *d*, Pearson's *r*), indicating how they were calculated

Our web collection on statistics for biologists contains articles on many of the points above.

Software and code

Policy information about availability of computer code

Data collection	Desmond (v. 7.0.128) by Schrödinger (Release 2022-2)<br>BRET data was collected on a BMG PHERAstar FSX 472-0142 with data processed in BMG MARS software 3.32.
Data analysis	Microsoft Excel for Mac (v. 16.82), Maestro (v. 13.2.128) by Schrödinger (Release 2022-2), GetContacts (https://getcontacts.github.io/), Graphpad Prism 9.0, CryoSPARC (v. 3.2.0), Relion (v. 3.1), crYOLO (v. 1.7.6), Sidesplitter (v. 1.2), Coot (v. 0.8.8) and Phenix (v. 1.19).

For manuscripts utilizing custom algorithms or software that are central to the research but not yet described in published literature, software must be made available to editors and reviewers. We strongly encourage code deposition in a community repository (e.g. GitHub). See the Nature Portfolio guidelines for submitting code & software for further information.

Data

Policy information about availability of data

All manuscripts must include a data availability statement. This statement should provide the following information, where applicable:

- Accession codes, unique identifiers, or web links for publicly available datasets
- A description of any restrictions on data availability
- For clinical datasets or third party data, please ensure that the statement adheres to our policy

The HU210/CB1/Gi1 cryo-EM structure and density are available in the Protein Data Bank under accession code PDB 9ERX [https://doi.org/10.2210/pdb9ERX/pdb].

The source data underlying Figures 2a-e and 6 (MS Excel), and initial and final configurations of one MD trajectory per ligand (PDB files), are provided as a Source Data file (zip archive).

Research involving human participants, their data, or biological material

Policy information about studies with human participants or human data. See also policy information about sex, gender (identity/presentation), and sexual orientation and race, ethnicity and racism.

Reporting on sex and gender	N/A
Reporting on race, ethnicity, or other socially relevant groupings	N/A
Population characteristics	N/A
Recruitment	N/A
Ethics oversight	N/A

Note that full information on the approval of the study protocol must also be provided in the manuscript.

Field-specific reporting

Please select the one below that is the best fit for your research. If you are not sure, read the appropriate sections before making your selection.

☒ Life sciences ☐ Behavioural & social sciences ☐ Ecological, evolutionary & environmental sciences

For a reference copy of the document with all sections, see nature.com/documents/nr-reporting-summary-flat.pdf

Life sciences study design

All studies must disclose on these points even when the disclosure is negative.

Sample size	No statistical tests were used to predetermine sample sizes. We used sample sizes commonly used in the literature in the field. Sample sizes were ndicated in the figure legends.
Data exclusions	No data was excluded from the analysis
Replication	Data are epresentative of three or more independent experiments as indicated in the figure legends. All attempts at replication were successful.
Randomization	All samples were randomly allocated to experimental groups. Each biological independent experiment was performed independently on a different day.
Blinding	Blinding was not applicable to this study, such as during in vitro experiments, where experimenters are required to know the conditions of each test well. Data collection and analysis wrer conducted independently by D Sykes & P Hompluem.

Reporting for specific materials, systems and methods

We require information from authors about some types of materials, experimental systems and methods used in many studies. Here, indicate whether each material, system or method listed is relevant to your study. If you are not sure if a list item applies to your research, read the appropriate section before selecting a response.

Materials & experimental systems

n/a	Involved in the study	
☒	☐	Antibodies
☐	☒	Eukaryotic cell lines
☒	☐	Palaeontology and archaeology
☒	☐	Animals and other organisms
☒	☐	Clinical data
☒	☐	Dual use research of concern
☒	☐	Plants

Methods

n/a	Involved in the study	
☒	☐	ChIP-seq
☒	☐	Flow cytometry
☒	☐	MRI-based neuroimaging

Eukaryotic cell lines

Policy information about cell lines and Sex and Gender in Research

Cell line source(s)	T-REx™-293 cells (R71007) were obtained from ThermoScientific.
Authentication	Stably transfected versions of T-Rex™-293 cell were validated for functional signalling responses using reference CB1R compounds.
Mycoplasma contamination	All cell lines tested negative for mycoplasma contamination.
Commonly misidentified lines (See ICLAC register)	No commonly misidentified cell lines were used in this study.

Plants

Seed stocks	N/A
Novel plant genotypes	N/A
Authentication	N/A
